# RNA-Seq Reveals OTA-Related Gene Transcriptional Changes in *Aspergillus carbonarius*

**DOI:** 10.1371/journal.pone.0147089

**Published:** 2016-01-14

**Authors:** Donato Gerin, Rita M. De Miccolis Angelini, Stefania Pollastro, Francesco Faretra

**Affiliations:** Department of Soil, Plant and Food Sciences, Section of Plant Pathology, University of Bari Aldo Moro, Bari, Italy; Universita degli Studi di Pisa, ITALY

## Abstract

Ochratoxin A (OTA) is a mycotoxin harmful for animals and humans. *Aspergillus carbonarius* is the main responsible for OTA contamination of grapes and derived products. Gene transcriptional profiling of 4 *A*. *carbonarius* strains was carried out by RNA-Seq analysis to study transcriptome changes associated with OTA production. By comparing OTA inducing (OTAI) *vs*. non-inducing (OTAN) cultural conditions, a total of 3,705 differentially expressed genes (DEGs) (fold change > |2| and FDR ≤ 0.05) were identified. Several genes involved in primary metabolic processes, with particular regard to carbohydrate and amino acid metabolisms, secondary metabolic processes, transport, response to stress and sporulation were up-regulated by OTAI conditions at all the analysed sampling times (4, 6 and 8 DAI) or starting from 6 DAI. Highly up-regulated DEGs encoding enzymes involved in biosynthesis of secondary metabolites, oxidoreductases, transporters and transcription factors were examined for their potential involvement in OTA biosynthesis and related metabolic pathways. Differential expression of genes encoding polyketide synthases (*pks*), non-ribosomal peptide synthetases (*nrps*) and chloroperoxidase (*cpo*) was validated by RT-qPCR. Among clusters of co-regulated genes involved in SM biosynthesis, one putative OTA-gene cluster, including both *pks* and *nrps* genes, was detected in the *A*. *carbonarius* genome.

## Introduction

Ochratoxin A (OTA) is a mycotoxin produced by fungal species belonging to the genera *Aspergillus* and *Penicillium*. It is responsible for several adverse effects on animals and humans, having neurotoxic, carcinogenic, immunotoxic, genotoxic, teratogenic properties, and is classified as possible human carcinogen (group 2B) by the International Agency for Research on Cancer [1]. OTA is a common contaminant of various foods and drinks [2,3,4,5,6], and was first reported in wine by Zimmerli and Dick [7]. After cereals, wine is the second source of daily OTA intake for humans [8]. *Aspergillus carbonarius* (Bainier) Thom is recognized as the main OTA-producing fungus on grapes and derived products [9,10,11].

The chemical structure of OTA, namely N-{[(3R)-5-chloro-8-hydroxy-3-methyl-1-oxo-3,4-dihydro-1H-isochromen-7-yl]carbonyl}-L-phenylalanine, consists of a pentaketide-derived dihydroisocoumarin moiety linked by an amide bond to a phenylalanine amino acid [12]. The chlorine atom attached to the dihydroisocoumarin aromatic ring confers a relatively high toxicity to the molecule in comparison to other ochratoxins [13]. The OTA biosynthetic pathway has not yet been fully clarified, even if many studies have been carried out in several OTA-producing fungi leading to the identification of key enzymes: a polyketide synthase (PKS) required for the biosynthesis of the dihydrocumarin’s precursor; a methyltransferase or a C-methylation (C-Met) domain included in the PKS, adding a methyl group to the C-7 of the newly formed molecule, and a P450 monooxygenase (CYP), oxidizing the methyl group to a carboxyl group; a non-ribosomal peptide synthetase (NRPS) catalyzing the formation of a peptide bond between the polyketide and a phenylalanine; and, finally, a chloroperoxidase adding a chlorine atom at the C-5 position [14].

Disruption of the *pks* gene in *Aspergillus ochraceus* proved its functional role in OTA biosynthesis [15]. A similar approach showed the involvement of the *aoks1* gene in *Aspergillus westerdijkiae* [16] and the *AcOTApks* gene in *A*. *carbonarius* [17]. The role of NRPS, previously reported in *Penicillium nordicum* (encoded by the *nps*PN gene) and *Penicillium verrucosum* (*nps*PV) [18], has been demonstrated in *A*. *carbonarius* (*AcOTAnrps*) by gene knockout [19].

Several techniques, such as AFLP-based transcript profiling (cDNA-AFLP) [20] and suppression subtractive hybridization (SSH) [21], have been used to identify genes differentially expressed in OTA producer and non-producer *A*. *carbonarius* strains. Recently, the complete genome sequences of an OTA non-producer *A*. *carbonarius* strain has been released [22]. However, the knowledge on OTA biosynthetic pathway in the fungus, its activation and regulation remains incomplete.

High-throughput Next Generation Sequencing (NGS) technologies make nowadays possible to carry out detailed transcriptomic profiling. In this study, we applied the NGS-based RNA-Seq method to carry out a global transcriptional analysis on 4 OTA-producer strains of *A*. *carbonarius* grown under inducing and non-inducing conditions. Differential gene expression analysis was aimed at improving knowledge on OTA-related gene expression and at identifying genes directly or indirectly related to OTA biosynthetic pathway.

## Materials and Methods

### *A*. *carbonarius* strains and growing conditions

The OTA-producer strains of *A*. *carbonarius* used in this study, AC49, AC66, AC67 and AC70, were obtained from naturally infected grape berries. All strains were grown on potato dextrose agar (PDA; infusion from 200 g peeled and sliced potatoes kept at 60°C for 1 h, 20 g dextrose, adjusted at pH 6.5, 20 g agar Oxoid no. 3, per litre) at 25°C in the dark for 5 days. Conidia of each strain were inoculated (1x10^6^ mL^-1^, final concentration) into 250 mL Erlenmeyer flasks containing 100 mL of minimal medium [MM; 10 mL solution A (10 g KH_2_PO_4_, 100 mL^−1^ water), 10 mL solution B (20 g NaNO_3_, 5 g KCl, 5 g MgSO_4_·7H_2_O, 0.1 g FeSO_4_, 100 mL^−1^ water), 1 mL of micronutritive solution [23], 20 g glucose, per litre] and incubated at 25°C in the dark, with or without shaking (150 rpm), corresponding to non-inducing (OTAN) or inducing (OTAI) conditions for OTA production, respectively.

Three replicated cultures were grown for each combination of strains and conditions. Culture broths for OTA quantification were obtained by filtration through Miracloth (Calbiochem, San Diego, California, USA) 0, 2, 4, 6 and 8 Days After Inoculation (DAI). The mycelium was collected at 4, 6 and 8 DAI, frozen in liquid nitrogen and stored at -80°C until use for RNA-Seq analysis.

### OTA Quantification

For HPLC analysis of OTA, aliquots (100 μL) of culture broths were injected into the chromatographic apparatus made up by an isocratic pump (HP 1100, Agilent Technologies, Santa Clara, California, USA) equipped with an injection valve (mod. 7125, Rheodyne, Cotati, California, USA), a fluorometric detector (HP 1100, λ_ex_ = 333 nm, λ_em_ = 460 nm) and a Chemstation Rev A.08.03 data system (Agilent Technologies). The analytical column was a reversed-phase Discovery C18 (15 cm x 4.6 mm, 5 mm particles) (Supelco, Bellefonte, Pennsylvania, USA) preceded by a SecurityGuard (Phenomenex, Torrance, California, USA). OTA in extracts was identified because having a retention time identical to that of the OTA standard (Supelco Sigma-Aldrich, St. Louis, Missouri, USA).

### RNA extraction, RNA-Seq library preparation and sequencing

For RNA-Seq analysis, pooled mycelia from 3 replicated cultures were used to prepare an RNA sample per each strain, growing condition and sampling time. Frozen mycelium (100 mg) was powdered in liquid nitrogen. Total RNA was extracted and purified by using RNeasy Plant Mini Kit (Qiagen, Milan, Italy), following the manufacturer's protocol. RNA quantity and quality were determined with a Nanodrop 2000 spectrophotometer (Thermo Fisher Scientific Inc., Wilmington, Delaware, USA) and a Bioanalyzer 2100 (Agilent Technologies, Santa Clara, CA, USA). cDNA libraries were prepared from 4 μg total RNA using TruSeq RNA Sample Preparation Kit v2 (Illumina, Inc., San Diego, California, USA) and validated according to Illumina’s low-throughput protocol. After normalization, cDNA libraries were pooled for multiplexing before loading onto a flow cell (8–9 samples per lane). The hybridization and cluster generation were performed on a cBot System using TruSeq SR Cluster Kit v3. Sequencing was performed on an Illumina HiScanSQ platform using TruSeq SBS kit v3 (Illumina, Inc.) to obtain Single Reads, 50 nt in length. Indexed raw sequencing reads from each library were de-multiplexed using the CASAVA v1.8 software (Illumina, Inc.).

### RNA-Seq data analysis

The quality of the raw sequence reads was checked using FastX-tools integrated into the Galaxy platform (https://usegalaxy.org/; [24]). Filtered reads from each sample were then separately aligned to the reference genome of *A*. *carbonarius* (ITEM 5010, assembly v3; DOE Joint Genome Institute, http://genome.jgi-psf.org/Aspca3/Aspca3.home.html), using the Q-Seq module of the ArrayStar software v. 5.0.0 (DNASTAR, Madison, WI, USA) with default mapping parameters, and used to estimate the abundance of 11,624 gene transcripts, measured as Reads Per Kilobase per Million mapped reads (RPKM) [25]. RPKM > 0.5 was used as cut-off for gene expression. A multiple correlation test (Cross-R^2^ statistical test, Q-Seq) on RPKM values for all pairwise combinations was performed for preliminary batch comparisons of replicates and experimental conditions.

Differential gene expression analysis was performed with the statistical R package DESeq (http://bioconductor.org/packages/2.12/bioc/html/DESeq.html) using the total number of sequence reads from individual samples mapped on annotated transcripts, and the 4 *A*. *carbonarius* strains as biological replicates. Fold Change (FC) was calculated comparing the RPKM expression values under OTAI *vs*. OTAN conditions; an expression value of 0.05 RPKM was imposed to non-expressed genes. False Discovery Rate (FDR) was determined and genes with FC > |2| and FDR ≤ 0.05 in at least one of the sampling times were considered as differentially expressed genes (DEGs) and submitted to functional analysis. The expression profiles of DEGs at different DAI were analysed by hierarchical clustering and heat map of expression values (T-MeV 4.9.0 software [26]).

### Functional analysis and clustering of DEGs

Blast2GO v2.6.4 [27] was used for functional annotation of *A*. *carbonarius* transcriptome, and *Aspergillus*-specific GO Slim (http://www.geneontology.org/ontology/subsets/goslim_aspergillus.obo) for summarising GO annotations. GO Enrichment analysis (Fisher's Exact Test) was used (*p*-value ≤ 0.05) to detect functional categories of biological processes and molecular functions over-represented, with statistical significance (FDR ≤ 0.05), in each DEG set as compared with the remaining non-differentially expressed genes (used as reference transcriptome). The distribution of GO terms in DEG sets was explored through Multilevel Pie charts.

The antiSMASH 2.0 platform (http://antismash.secondarymetabolites.org/ [28]) was used to predict gene clusters involved in biosynthetic pathways of secondary metabolites (SMs) in the *A*. *carbonarius* masked scaffolds and to perform a ClusterBlast analysis on available fungal genome sequences (http://genome.jgi-psf.org/programs/fungi/index.jsf). Co-expressed genes (CEGs) were identified using the quality threshold (QT) clustering (Pearson correlation ≥ 0.75, minimum 4 genes per cluster) in T-MeV 4.9.0 software and clustered in groups showing similar expression profiles and including maximum one non-correlating gene in between them. The average RPKM values of the CEGs included in each putative gene clusters were used to explore their co-regulation through correlation analysis.

### Validation of RNA-Seq analysis by RT-qPCR

RT-qPCR analysis was carried out according to MIQE guidelines [29] to validate RNA-Seq results for ten selected DEGs. Total RNA was extracted from 2 *A*. *carbonarius* strains, AC49 and AC67, used as biological replicates, using TRI Reagent (Sigma-Aldrich) according to the manufacturer’s instructions. All primer pairs were designed with the Primer3 software (http://frodo.wi.mit.edu/primer3/ [30]) with the forward ones designed on the exon-junction sites of the target gene to amplify only cDNA and not possible contaminant genomic DNA (S1 Table). First strand cDNA was synthesized from 2 μg of RNA using M-MLV reverse transcriptase (Life Technologies, Milan, Italy) and random primers in a volume of 20 μL, according to the manufacturer’s instructions. qPCR was performed in a CFX96^TM^ Real-Time PCR Detection System (Bio-Rad Laboratories, Hercules CA, USA) in a volume of 25 μL containing 12.5 μL of iQ SYBR Green Super Mix (Bio-Rad Laboratories), 0.5 μM of each primer and 1 μL of the reverse transcription reaction. The conditions for amplification were as follows: 3 min denaturation at 95°C followed by 35 cycles of 95°C for 10 s and 60°C for 45 s. The absence of unwanted products was assessed though melting curve from 60°C to 95°C with 10-sec steps of 0.5°C increments. All samples were analysed in duplicate. Genes encoding β-tubulin (*β-tub*; ID 202852), calmodulin (*cal*; ID 205510), and ubiquitin-conjugating enzyme (*ubc*; ID 393986) from the *A*. *carbonarius* genome were selected as candidate references and BestKeeper algorithm was used to evaluate their gene expression stability [31]. Relative gene expression was calculated using CFX Manager Software (Bio-Rad Laboratories) and the 2^-ΔΔCT^ method [32].

## Results

### OTA production under inducing and non-inducing conditions

The production of OTA was favoured in static cultures in MM (OTAI) and prevented in shaken cultures in MM (OTAN). Under OTAN conditions, OTA concentrations decreased or remained stable from 0 to 8 DAI. Under OTAI conditions, OTA concentrations increased from 2 to 8 DAI with a strain-dependent variation. The strain AC49 was the best OTA-producer followed, in the order, by AC70, AC67 and AC66 (Table 1).

**Table 1:** OTA concentrations (μg L^-1^) at different Days After Inoculation (DAI) in cultural broth of 4 *A*. *carbonarius* strains grown in liquid minimal medium (MM) under OTA inducing (OTAI, static culture) and non-inducing (OTAN, shaken culture) conditions. Data are the mean values of 3 replicates and their standard errors.

Strain	Cultural condition	DAI				
		0	2	4	6	8
AC49	OTAI	171.5±4.4	174.9±5.7	221.4±10.2	754.3±45.1	1,603.4±212.5
AC67	OTAI	115.7±3.9	111.1±2.6	143.7±6.8	479.7±92.8	558.5±129.7
AC66	OTAI	19.6±0.8	19.6±0.3	19.8±1.0	150.8±40.4	425.3±97.5
AC70	OTAI	5.8±0.2	5.9±0.2	6.1±0.2	177.6±69.7	1,343.9±180.8
AC49	OTAN	180.6±7.2	144.0±10.1	86.4±9.6	80.7±12.2	80.9±27.2
AC67	OTAN	113.6±5.0	96.4±11.7	50.2±2.1	47.3±3.5	36.6±3.8
AC66	OTAN	19.8±1.0	20.9±0.5	15.7±2.9	15.6±4.3	20.3±1.7
AC70	OTAN	5.5±0.3	6.2±0.2	7.0±0.3	7.0±0.5	5.5±1.9

### RNA-Seq analysis

A summary of RNA-Seq data is reported in S2 Table. A total of 255,290,345 high-quality (QS≥30) short-sequence single reads (50 bp), yielding approximately 13 Gb of transcriptomic sequence data, was obtained from all the sequenced samples and deposited in the NCBI Sequence Read Archive (SRA) database under study accession number SRP059422. Approximately 82% of short reads, more than 6.6 million qualified reads per each biological replicate, were successfully mapped on annotated gene transcripts of the *A*. *carbonarius* genome, corresponding to a coverage of at least 14X of the fungal transcriptome. Average GC content in the sequenced transcriptomes was 54%. The number of total mapped reads increased (237,697,062, *i*.*e*. 93%) when they were aligned to masked scaffolds of the assembled *A*. *carbonarius* genome. No significant differences were observed in terms of total number of aligned reads between the tested strains (AC49, AC66, AC67 and AC70), conditions (OTAI and OTAN) or sampling times (4, 6 and 8 DAI). Most of the mapped reads were uniquely assigned to transcript sequences (54–73%), while the remaining reads were unmapped (12–34%) or showed multi-position matches (12–16%). A total of 10,479 and 10,257 genes, out of 11,624 predicted genes in the *A*. *carbonarius* genome, were expressed under OTAI and OTAN conditions, respectively. A high Person’s correlation coefficient (R^2^) (0.86) was observed between RPKM values of the 4 *A*. *carbonarius* strains, used as biological replicates, indicating that the approach was appropriate for further analysis (S3 Table). A high correlation (average R^2^ ≥ 0.82) was observed between sampling times in both OTAI or OTAN conditions; lower R^2^ values were observed when the two growing conditions were compared at 4 (average R^2^ = 0.82), 6 (R^2^ = 0.72) and 8 DAI (R^2^ = 0.77). Hence, sampling time caused fewer transcriptional changes than growing conditions.

### Differential gene expression and functional analysis

**Fig 1:**
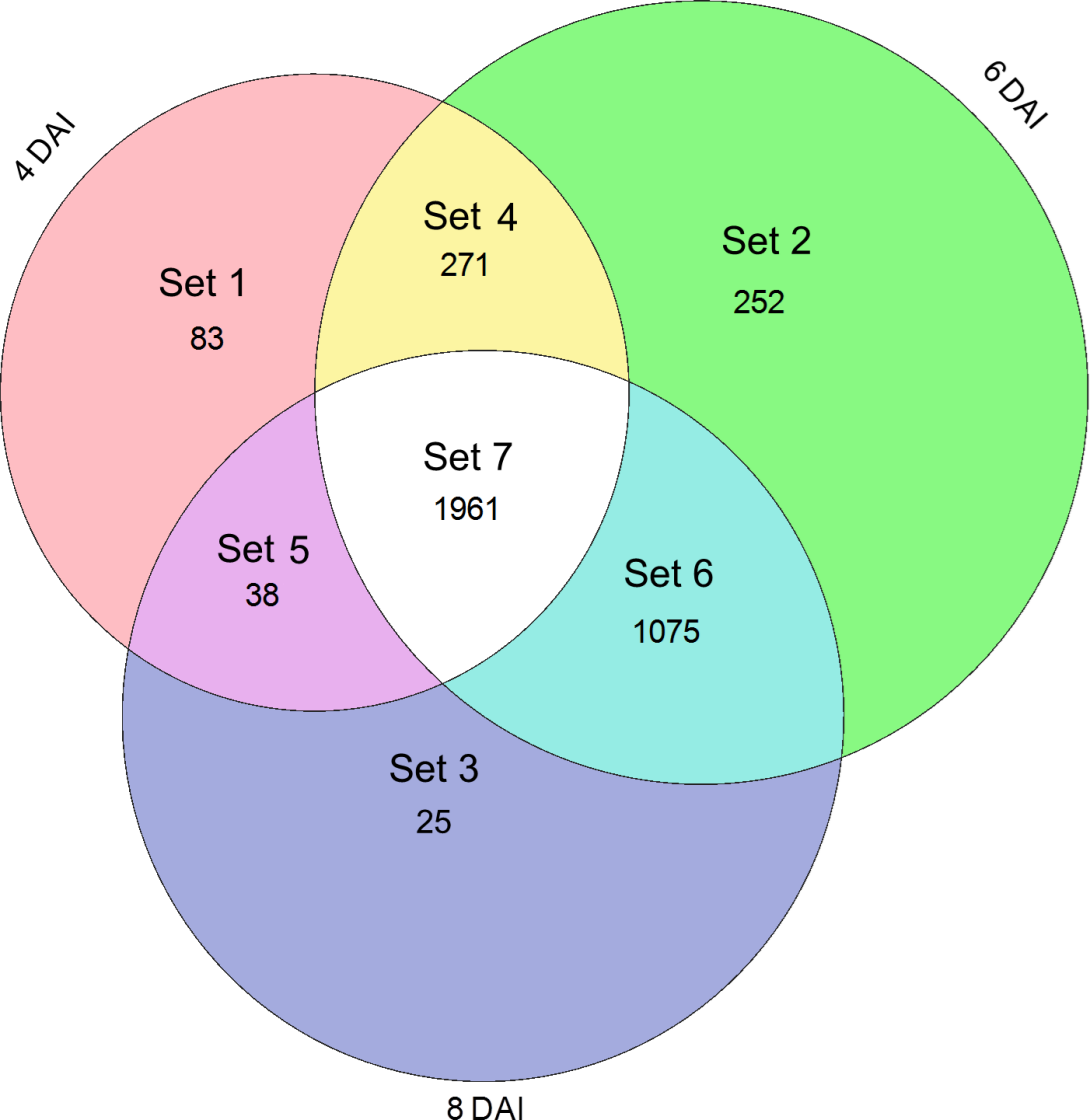
Venn diagram of DEGs (FC >|2|) comparing OTAI *vs*. OTAN conditions at 4, 6 and 8 DAI.

**Fig 2:**
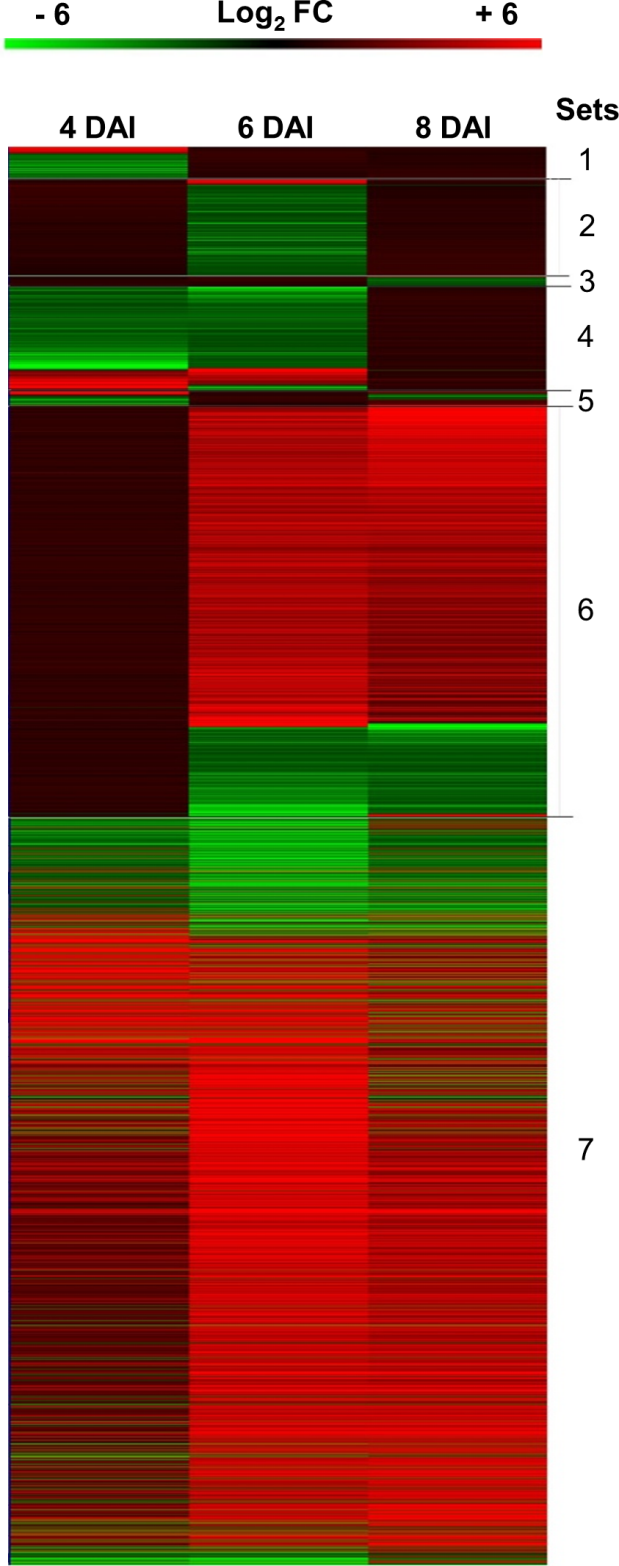
Heat-map showing expression profiles of DEG sets identified by the Venn diagram.

A total of 3,705 differentially expressed genes (DEGs; FC > |2|, FDR ≤ 0.05) were identified comparing OTAI *vs*. OTAN conditions and submitted to clustering and functional analysis. Relatively few genes (0.7–6.8%) were modulated only at single sampling times (Sets 1–3), whereas the majority of DEGs were modulated at 6 and 8 DAI (29%, Set 6) or at all times (53%, Set 7) (Fig 1). The expression profiles of DEG sets are reported in Fig 2. Sets 1 to 5 contained more down-regulated than up-regulated DEGs; as opposite, the Sets 6 and 7 contained prevalently up-regulated DEGs.

Distribution of GO-terms among up- and down-regulated DEGs is shown in S1 Fig. Some functional categories were related to both primary and secondary metabolic processes, fungal growth, cell cycle and reproduction. The term asexual sporulation was strictly associated with up-regulated DEGs, while sexual sporulation was prevalently associated with down-regulated DEGs. GO-terms, such as vesicle-mediated transport, lipid metabolic processes, protein catabolic process and response to stress, were more frequently associated to up- than to down-regulated DEGs.

**Fig 3:**
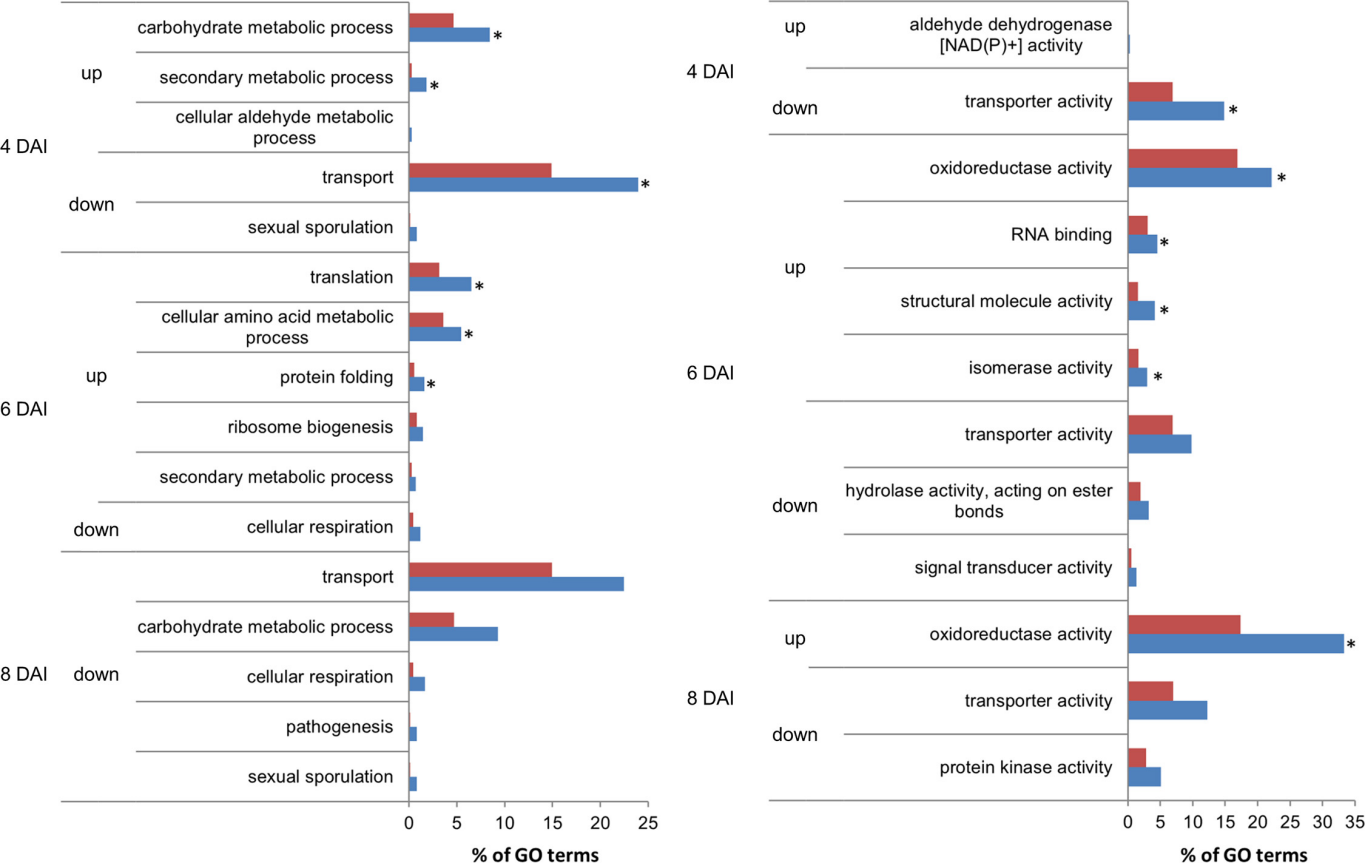
Enrichment analysis for biological process (a) and molecular function (b) for genes up- or down-regulated at different DAI (Days After Inoculation). Asterisks indicate GO categories over-represented (FDR ≤ 0.05) among DEGs (blue) compared to the reference *A*. *carbonarius* transcriptome (red).

Enrichment analysis for GO terms was carried out in order to determine processes and functions over-represented in DEG sets as compared to the reference transcriptome (Fig 3 and S4 Table). Among up-regulated DEGs, processes related to secondary metabolism (1.8%) and carbohydrate metabolism (8.4%) were significantly over-represented in OTAI conditions at 4 DAI; secondary metabolic processes were more represented, although with no statistical significance, at 6 DAI (0.7%), along with terms related to amino acid and protein metabolism, such as cellular amino acid metabolic process (5.5%), translation (6.5%), protein folding (1.6%) and ribosome biogenesis (1.5%). Among down-regulated DEGs, over-represented process categories were transport (22.5–24.0%) and sexual sporulation (0.8%) at 4 and 8 DAI, cellular respiration (1.2–1.7%) at 6 and 8 DAI, carbohydrate metabolic process (9.3%) and pathogenesis (0.8%) at 8 DAI. In particular, secondary metabolic processes were associated to DEGs such as *pks*, *nrps*, phenylalanine ammonia-lyase (*PAL*) and genes related to pigment biosynthesis and antibiotic biosynthetic pathways. Processes of carbohydrate metabolism were associated to up-regulated DEGs involved in catabolism, *e*.*g*. hydrolases, and to down-regulated DEGs involved in anabolism, *e*.*g*. synthases and transferases.

As to molecular functions is concerned, among up-regulated DEGs, oxidoreductase activity was significantly over-represented at 6 and 8 DAI (22.2–33.3%); additional terms at 6 DAI were RNA-binding (4.5%), structural molecular activity (4.2%) and isomerase activity (3.0%). Among down-regulated DEGs, over-represented functions were transporters (all times), signal transducers, hydrolases acting on ester bonds (6 DAI) and protein kinase activities (8 DAI).

Eight-hundred-ninety highly up-regulated (FC > 8, FDR ≤ 0.05) DEGs were selected. K-means clustering grouped them into 4 clusters sharing similar temporal expression patterns (S2 Fig). In this sets, we detected, prevalently in the clusters b and d, 151 DEGs of potential interest for their functions and temporal expression profiles coherent with OTA production (Table 2).

**Table 2:** Selected *A*. *carbonarius* genes highly up-regulated under OTAI *vs*. OTAN conditions.

Transcript ID*	Functional category	FC**		
		4 DAI	6 DAI	8 DAI
*Secondary metabolic processes*				
5570/5640	polyketide synthase	6.7	**59.0**	**59.0**
56260	polyketide synthase	1.5	**382.9**	235.2
172075	polyketide synthase (*Alb1*)	**36.0**	10.7	6.2
505925	polyketide synthase (*Acpks*)	**40.9**	**18.6**	11.9
173482	polyketide synthase (*AcOTApks*)	2.1	**6.3**	2.1
132610	non-ribosomal peptide synthetase (*AcOTAnps*)	**38.5**	**70.9**	6.9
204544	non-ribosomal peptide synthetase	**49.6**	**70.3**	**41.5**
206989	non-ribosomal peptide synthetase	**48.6**	**80.8**	**39.2**
505182	non-ribosomal peptide synthetase	1.1	**7.1**	**10.2**
208824	non-ribosomal peptide synthetase-like enzyme	**8.5**	**98.9**	**106.6**
209543	radH flavin-dependent halogenase	**39.2**	**247.5**	24.6
205535	cercosporin toxin biosynthesis protein	6.5	**29.9**	**38.4**
127829	steroid monooxygenase	5.5	**21.9**	7.7
10909	salicylate hydroxylase	**9.5**	7.5	14.2
208828	GPI anchored dioxygenase	**40.3**	**43.7**	**16.7**
205273	dienelactone hydrolase family protein (HDL)	1.1	**10.9**	**12.1**
397762	dienelactone hydrolase family protein (HDL)	2.8	**13.5**	19.2
158065	oxalacetate acetylhydrolase	3.0	**11.0**	**12.1**
512610	sesquiterpene cyclase	12.7	**49.0**	14.2
132163	Rab geranylgeranyl transferase, beta subunit	3.5	**10.9**	10.3
209742	conidial pigment biosynthesis oxidase (*Abr1*)/brown 1	**23.7**	**17.6**	**11.3**
8073	pigment biosynthesis protein (*Ayg1*)	**66.2**	**12.6**	6.8
5560/405938	conidial pigment biosynthesis scytalone dehydratase (*Arp1*)	6.2	**47.1**	**48.0**
*Transport*				
209684	general substrate transporter-like protein	**14.6**	**45.1**	**26.4**
173073	efflux pump antibiotic resistance protein	**52.4**	**33.7**	**25.6**
209281	efflux pump antibiotic resistance protein	1.5	**9.1**	8.8
173225	MFS toxin efflux pump (*aflT*)	**13.8**	5.1	2.2
49247/49992	MFS transporter	**143.8**	**2,102**	**2,257**
130550/130610	MFS transporter	7.1	**29.5**	**94.3**
173694	MFS transporter	2.5	**11.9**	9.8
209285	MFS transporter	**35.5**	**23.7**	**12.2**
135554	MFS transporter (FLU1)	6.8	**12.2**	5.7
211441	ABC drug exporter (*AtrF*)	2.9	6.0	**14.5**
171384	ABC transporter	3.9	**46.5**	**27.9**
177479	Aquaporin	1.3	**12.3**	**11.8**
39367	dynamin family protein	**107.0**	**507.4**	**190.5**
204836	galactose-proton symporter	5.3	**14.1**	8.3
132418	formate/nitrite family transporter	5.5	**22.0**	**14.7**
392642	H+/nucleoside cotransporter	**10.3**	**14.0**	7.5
126007	purine-cytosine permease	**19.3**	3.6	1.1
125662	high affinity methionine permease	**17.9**	**18.5**	**28.3**
*Fungal growth and asexual sporulation*				
396337	solid-state culture specific protein	**8.2**	**42.2**	**25.6**
205578	plasma membrane protein Pth11-like protein	**18.5**	**15.8**	7.0
211206	cell wall protein	4.8	**10.8**	**9.9**
131880	cell wall protein	**56.7**	**11.5**	4.5
208632	F5/8 type C domain protein	**384.2**	**19.1**	9.0
130752	conidiation-specific protein 10	**16.1**	**27.3**	**20.5**
205405	hydrophobic surface binding protein A	**8.1**	8.2	4.8
208459	conidial hydrophobin Hyp1/(*RodA*)	**446.5**	**11.1**	5.4
205575	hydrophobin/(*RodB*)	**19.0**	**45.2**	12.7
130776	small S protein-like	**12.8**	**26.5**	**17.7**
506080	extracellular matrix protein	**44.1**	**11.9**	5.9
151218	NACHT domain protein	**19.0**	**15.6**	5.3
156387	NACHT domain protein	**24.6**	**28.0**	**19.8**
*Metabolic processes of carbohydrates*, *lipids*, *amino acids*, *proteins and other macromolecules*				
212042	beta-1,6-glucanase	**17.4**	**13.6**	**10.9**
510653	exo-beta-1,3-glucanase	**14.5**	**14.5**	10.5
167755	glucose dehydrogenase	**8.0**	7.5	2.6
38981	alpha-1,2-mannosidase	**15.5**	**8.6**	7.2
517301	cell wall glycosyl hydrolase	**26.6**	**11.6**	8.5
174174	polygalacturonase, putative	1.6	6.4	**20.5**
206965	Chitinase	4.5	**11.1**	5.1
157125	extracellular serine-rich protein	**10.7**	7.2	6.2
135028	Lipase	**9.0**	**29.7**	**32.4**
133260	Cholinesterase	**10.0**	**125.0**	**146.5**
510672	triacylglycerol lipase	**8.2**	**10.3**	**15.5**
132362	Carboxylesterase	3.0	**14.4**	**22.9**
46975	dihydrodipicolinate synthetase family protein	1.2	**24.1**	**25.8**
204357	3’-phosphoadenosine-5’-phosphosulfate reductase	4.0	**11.3**	**13.1**
505924	glucose-methanol-choline oxidoreductase	**66.3**	**320.6**	**361.9**
7736	acetylglutamate kinases	**4.4**	4.1	4.1
206969	N-acetyltransferase	**27.0**	**23.6**	20.7
154116	acetylglutamate kinase	**15.1**	**14.4**	**13.7**
399389	aminotransferase class-III	4.8	**46.5**	23.0
212184	NADP-specific glutamate dehydrogenase	**8.2**	6.8	6.9
507488	proline oxidase (Put1)	16.2	**34.0**	27.8
518143	Aspartokinase	4.6	**8.6**	**9.5**
507815	poly(aspartic acid) hydrolase	4.4	**13.9**	**11.3**
507808	X-Pro dipeptidyl-peptidase (S15 family)	**26.7**	**30.2**	**14.9**
6644	matrix metalloproteinase-11	**11.5**	**37.2**	**39.1**
134932	nucleoside-diphosphate-sugar epimerase	**9.1**	**10.4**	**12.3**
211990	nucleoside-diphosphate-sugar epimerase	4.7	**13.8**	3.7
210550	nucleoside-diphosphate-sugar epimerase	2.6	**8.5**	7.0
40537	UDP-glucose 4-epimerase	**9.7**	**9.0**	**9.5**
202711	RNAse III	**15.3**	**55.2**	**30.6**
208875	uracil-DNA glycosylase	2.2	**12.4**	7.1
204318	metal dependent phosphohydrolase	1.6	**9.4**	5.4
205142	IMP cyclohydrolase	2.6	**8.3**	6.0
208869	nucleoside diphosphate kinase	3.2	**19.4**	**12.8**
*Transferase activity*				
510988	UMTA methyltransferase family protein (MT)	**122.6**	**61.6**	**41.6**
56226	O-methyltransferase (MT)	1.0	**207.0**	139.1
133219	O-methyltransferase (MT)	12.6	**519.2**	31.5
131073	O-methyltransferase (MT)	**75.2**	**38.3**	**17.9**
50066	O-methyltransferase (MT)	**26.8**	**10.0**	6.0
492	O-methyltransferase (MT)	5.1	**15.8**	19.2
504341	O-methyltransferase (MT)	1.8	**10.7**	9.5
51018	Acyltransferase	6.5	**8.1**	10.6
*Response to stress*				
37752	Catalase	**10.4**	**32.4**	**17.6**
131650/516443	Catalase	**38.5**	**83.8**	**52.6**
9402	Catalase	**23.5**	**224.5**	**77.9**
518556	spore-specific catalase A (*AocatA*)	3.2	**21.1**	12.0
53994	stress response protein (*Rds1*)	**14.3**	**20.0**	7.3
*Oxidation-reduction processes*				
212238	chloroperoxidase (CPO)	**74.6**	**13.2**	7.2
399443	benzoate 4-monooxygenase cytochrome P450 (CYP)	2.8	**219.8**	22.5
208126	cytochrome P450 monooxygenase (CYP)	**63.5**	**15.2**	6.7
392816	cytochrome P450 monooxygenase (CYP)	3.4	**9.2**	13.4
517149	cytochrome P450 monooxygenase (CYP)	**38.2**	**382.2**	27.1
153077	cytochrome P450 monooxygenase (CYP)	1.2	**9.1**	**10.7**
53587	cytochrome P450 monooxygenase (CYP)	**8.7**	**12.5**	**9.4**
178693	cytochrome P450 monooxygenase (CYP)	2.9	**14.6**	**13.5**
516985	cytochrome P450 monooxygenase (CYP)	**22.5**	**68.0**	82.9
166660	cytochrome P450 monooxygenase (CYP)	**56.5**	**46.2**	**33.8**
56024	monooxigenase (MOX)	1.4	**533.5**	216.6
155617	flavin-containing monooxygenase (MOX)	**35.2**	**16.3**	7.4
45176	FAD-dependent monooxygenase (MOX)	**48.4**	**27.3**	4.6
206523	FAD-dependent monooxygenase (MOX)	**14.1**	**78.5**	**31.7**
53354	FAD dependent oxidoreductase	**181.0**	**47.0**	**43.6**
206408	FAD binding oxidoreductase	**33.5**	**29.2**	**23.3**
154209	aldo-keto reductase	**12.5**	**213.9**	**134.2**
204540	sulfite reductase	5.8	**9.1**	8.3
208167	NADPH-dependent FMN reductase	1.7	**9.0**	7.8
209570	amid-like NADH oxidoreductase	**21.2**	**30.0**	**9.7**
505939	pyridine nucleotide-disulfide oxidoreductase	**30.7**	3.4	4.8
207168	UDP-glucose dehydrogenase (DHO)	2.2	6.4	**8.7**
204657	alcohol dehydrogenase (DHO)	1.1	**11.2**	**18.5**
179224	short-chain dehydrogenase/reductase (DHO)	1.9	**9.3**	**11.2**
125746	short-chain dehydrogenase/reductase (DHO)	3.5	**9.9**	7.4
139967	retinol dehydrogenase 12	**10.7**	**27.5**	**10.8**
519260	multicopper oxidase	4.2	**194.7**	**325.3**
*Regulation*				
202814	fungal specific transcription factor	**21.4**	**8.8**	5.1
212444	C2H2 type conidiation transcription factor (*BrlA*)	**25.4**	3.9	3.7
400774	Zn(II)2Cys6 transcription factor	3.5	**9.4**	6.2
209691	plasma membrane ATPase proteolipid	5.7	**8.5**	6.4
206402	SesB-related regulatory protein	**39.2**	**74.2**	**74.8**
135080	BTB/POZ domain-containing protein	**11.1**	**16.0**	**17.9**
175588	protein phosphatase type 1 complex subunit Hex2/Reg1	**9.5**	**9.9**	4.9
178837	NmrA-like family protein	**105.3**	**99.4**	**56.8**
209900	NmrA-like family protein	**10.9**	**35.3**	**21.1**
*Protein folding*				
130540/130601	chaperone protein HSP31	1.2	**11.3**	8.2
*Signal transduction*				
203881	protein kinase domain-containing protein	**11.3**	**17.5**	**13.3**
519130	Ser/Thr protein phosphatase	**28.4**	**89.3**	**84.2**
*Transcription*				
209896	Nucleotidyltransferase	**107.4**	**24.1**	10.7
142425	WD repeat protein	1.6	6.1	**10.0**
*Translation*				
404332	translation elongation factor EF-1, α subunit	1.7	**8.6**	7.0
208755	tryptophanyl-tRNA synthetase	2.8	**13.9**	9.8
*Ribosome biogenesis*				
211752	snRNP and snoRNP protein	2.5	**8.8**	**9.2**

*Transcript identification number (http://genome.jgi-psf.org/Aspca3/Aspca3.home.html).

**Figures in bold were up-regulated with statistically significance (FDR ≤ 0.05).

Five *pks* genes and 5 *nrps* genes, coding for enzymes involved in biosynthesis of secondary metabolites (SMs), were identified. In particular, the 2 *pks* DEGs 5570 and 5640 were identical sequences included in a ~200 kb repeated genomic region. The *pks* DEG 505925 contained the partial *Acpks* gene sequence [33] and the DEG 173482 corresponded to the *AcOTApks* gene of *A*. *carbonarius*. The *nrps* DEG 132610 corresponded to the *AcOTAnps* gene of the same fungus. Additional DEGs were putatively involved in biosynthesis of mycotoxins and other SMs, such as chloroperoxidases (CPO) (1), cytochrome P450 monooxygenases (CYP) (9), monooxygenases (MOX) (4), dehydrogenases (DHO) (4), hydrolases (HDL) (2) and methyltransferases (MT) (7). Moreover, the DEG 158065 was homologous to the *A*. *niger ANI_1_92174* gene encoding oxalacetate acetylhydrolase, the enzyme converting oxalacetate to oxalate and acetate, which is a potential source of polyketide precursors.

The DEGs 172075, 8073, 209742 and 5560/405938, homologous to the *A*. *fumigatus* genes involved in melanin biosynthesis *Alb1*, *Ayg1*, *Abr1* and *Arp1*, respectively, showed an early up-regulation. Unlikely *A*. *fumigatus*, in which the four genes are in a cluster, the homologous sequences in *A*. *carbonarius* were located in different genomic regions. Moreover, 2 DEGs (208459 and 205575) encoding hydrophobins, homologous to the *A*. *fumigatus RodA* and *RodB* genes, and the DEG 212444, homologous to the *brlA* gene of *Aspergillus* species, encoding a transcription factor (C_2_H_2_ type) activating genes involved in asexual reproduction, were detected.

Three DEGs (37752, 131650/516443 and 9402) encoded catalases involved in stress response and 1 (518556) was homologous to the *A*. *ochraceus AocatA* gene coding for a spore-specific catalase involved in the maintenance of cellular redox balance.

Eight DEGs involved in transport functions were membrane transporters belonging to the Major Facilitator Superfamily (MFS). They included 2 identical sequences (49247 and 49992), the DEG 173225, homologous to the *aflT* gene in the aflatoxin biosynthesis gene cluster of *Aspergillus parasiticus* and *Aspergillus flavus*, and the DEG 135554, corresponding to the FLU1 MFS transporter active in *A*. *carbonarius* OTA-producer strains. The ABC transporter 211441 was homologous to the *A*. *fumigatus atrF* gene causing multidrug resistance and toxicant extrusion.

Several DEGs were involved in amino acid metabolism, such as 399389 (aminotransferase class-III), 212184 (NADP-specific glutamate dehydrogenase) and 507488 (proline oxidase, Put1), catalysing catabolic reactions leading to glutamate production, and 206969 (N-acetyltransferase), 154116 and 7736 (acetylglutamate kinases), involved in the conversion of glutamate to ornithine.

### Validation of RNA-Seq results by RT-qPCR

**Fig 4:**
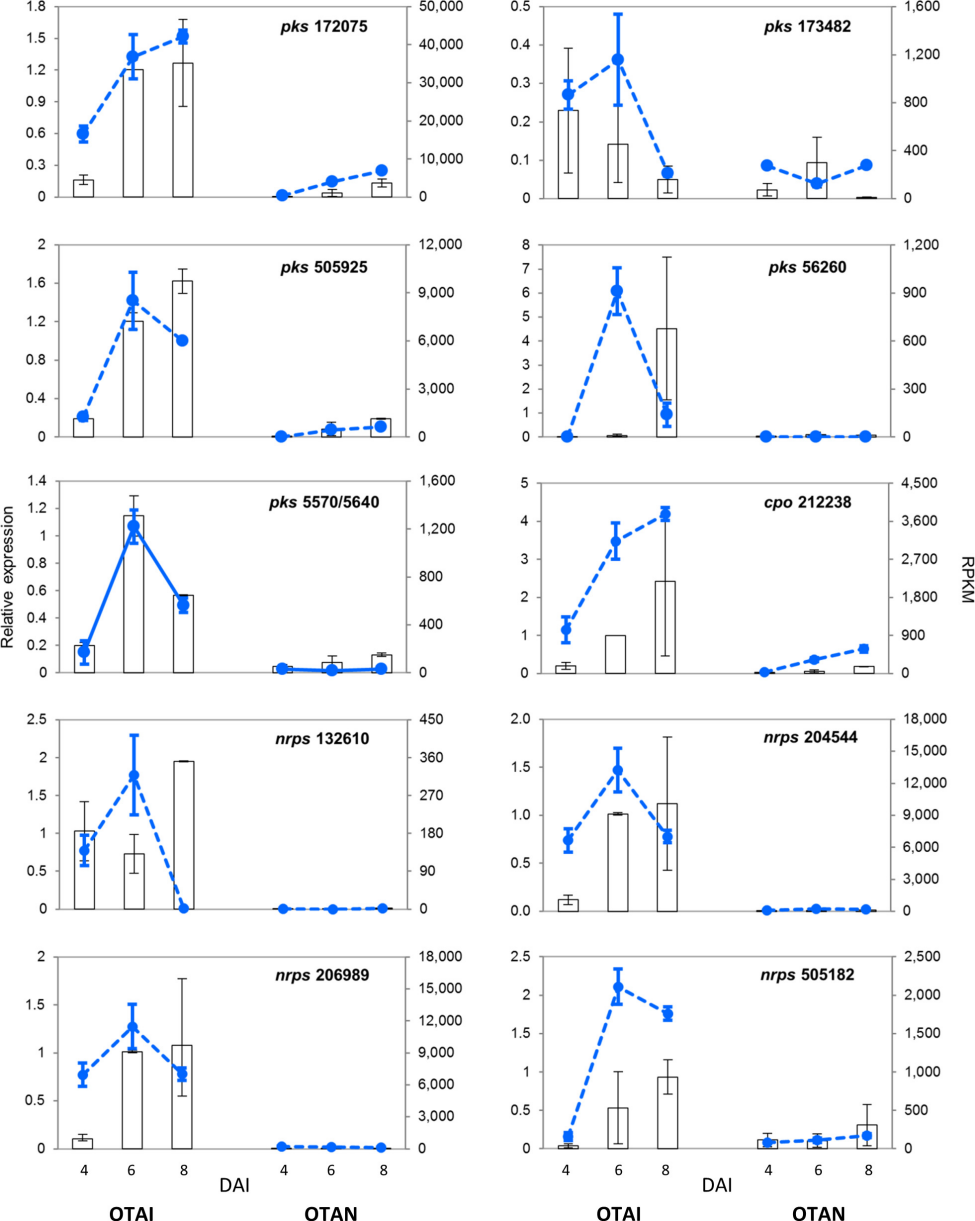
Comparison of RT-qPCR (dotted lines) and RNA-Seq (histograms) results for the *nrps*, *pks and cpo* genes up-regulated under OTAI conditions. Figures are the mean values of 2 biological replicates (strains AC49 and AC67) and bars represent standard error.

RT-qPCR was performed on selected DEGs: 5 *pks* (5570/5640, 56260, 172075, 173482 and 505925), 4 *nrps* (132610, 204544, 206989 and 505182) and 1 *cpo* (212238) DEGs. Three genes, *β-tub*, *cal* and *ubc*, were tested as candidate reference genes. The *ubc* gene showed the highest expression stability, according to the BestKeeper algorithm (*p*-value = 0.02; SD = 0.38; CV = 2.24), under the adopted experimental conditions, and it was hence used as reference gene. Gene expression patterns of the selected DEGS, assessed at 4, 6 and 8 DAI by RT-qPCR, were consistent with those obtained by RNA-Seq (Fig 4), confirming the reliability and accuracy of the NGS analysis.

### Co-expression analysis and putative OTA gene cluster

AntiSMASH 2.0 predicted 51 gene clusters involved in the synthesis of SMs in the *A*. *carbonarius* genome. Nine predicted clusters included a *pks gene*, 13 an *nrps* gene, 4 an hybrid *pks*/*nrps* gene, 1 included both *pks* and *nrps* genes, 5 were likely involved in terpene biosynthesis and 20 in other classes of end products (S5 Table).

Fifteen of 51 predicted gene clusters included 4 to 8 CEGs. Only the putative gene cluster 17 was down-regulated under OTAN conditions, while all the other putative gene clusters were up-regulated. These were divided in 2 groups on the ground of their expression profile: i) clusters with expression values increasing from 4 to 8 DAI (clusters 4, 13, 14, 19, 22, 37) and ii) clusters with expression values increasing from 4 to 6 DAI and decreasing later (24, 25, 26, 32, 38, 40, 42, 48) (S5 Table).

The putative gene cluster 38 displayed high homology with the OTA-gene cluster of *A*. *niger* and was made up by 6 CEGs. It contained both the *nrps* (132610) and *pks* (173482) DEGs up-regulated under OTAI conditions which were homologous to the *pks* and *nrps* genes playing a role in OTA biosynthesis in other *Aspergillus* species and in *P*. *nordicum*. In particular, the protein encoded by the *pks* gene in the cluster 4 showed 64% and 74% identities with the homologous proteins of *A*. *ochraceus* (AAP32477.1) and *A*. *niger* (CAK42679.1), respectively, and lower identities with OTA PKSs of *A*. *westerdijkiae* (AAT92023.1) (37%) and *P*. *nordicum* (AAP33839.2) (34%); while the *nrps* gene in the same cluster codes for a protein homologous to OTA NRPSs of *A*. *niger* (CAK42678.1) (60%) and *P*. *nordicum* (AAS98174.1) (39%). The cluster also included the cytochrome P450 monooxygenase (*cyp*) DEG 517149, showing homology in BlastP search (66% identity) with the *p450-B03* gene of *A*. *ochraceus* suggested to be involved in OTA biosynthesis, in addition to a flavin-containing monooxygenase (*fmo*) DEG (209543), a hypothetical protein DEG (209537) and a bZIP type transcription factor (*bZIP*) DEG (7821) (S5 Table).

Most of the putative gene clusters, with the exception of the clusters 22, 32 and 42, were not detected in other species belonging to the *Aspergillus* section *Nigri*. The cluster 40, containing a *nrps* gene and the cluster 42, containing a *pks* gene, along with the clusters 24 and 48 showed to be significantly co-regulated (R^2^≥0.75) with the putative OTA gene cluster (38) (S6 Table).

## Discussion and Conclusions

*A*. *carbonarius* is the main OTA-producing fungus in grape and derived products, especially in the Mediterranean areas [2,10]. OTA is a secondary metabolite derived from a polyketide precursor which is enzimatically modified to produce a chlorinated isocoumarin derivative linked to the amino acid L-phenylalanine. The OTA biosynthetic pathway has not yet been completely clarified in *A*. *carbonarius* and other *Aspergillus* species, and only few structural and regulatory genes have been suggested or proved to be involved [15,16,17,19,34].

In the present study, we applied RNA-Seq to study whole transcriptional changes associated to OTA production in 4 *A*. *carbonarius* strains grown under OTAI and OTAN conditions, aiming at getting a more global and accurate picture of OTA biosynthetic pathway and regulatory mechanisms. The same approach has been successfully applied to investigate several aspects in other *Aspergillus* species (*e*.*g*., [35]).

About 86% of 50-bp reads generated from RNA-Seq mapped to the reference *A*. *carbonarius* genome and allowed us to quantify transcript abundance for over 93% of annotated genes. About 13% of reads were within intergenic or intronic genomic regions and ∼14% were unmapped, presumably because derived from till unrecognized transcripts or transcript isoforms, polymorphic regions or sequence aberrations in the genomes of the tested isolates [36]. The large RNA-Seq data sets obtained (∼13 Gb) might be useful for improving the quality of annotation of the *A*. *carbonarius* genome and for getting deeper insights into other growing-dependent biological processes, such as secondary metabolism and fungal development (*e*.*g*., conidiogenesis).

Culture media and growing conditions greatly affect fungal growth and metabolite production. Several authors reported that static cultures in MM or other media induce OTA production in *Aspergillus* and *Penicillium* species ([18,33] [15]). We confirmed that OTA production in *A*. *carbonarius* is induced in static cultures and prevented in shaken cultures in MM. Four *A*. *carbonarius* strains were grown in the same medium to compare their transcriptomes under these two basic growing conditions and avoiding interferences of other nutritional and environmental factors. Both RNA-Seq analysis and OTA quantification in cultural broths were carried out at different sampling times, in order to investigate on relationships between gene transcription and OTA biosynthesis and secretion.

OTAI conditions caused a significant transcriptional changes in a large set of genes. Analysis of transcriptional profiles showed that, generally, the genes early modulated, until 6 DAI, were down-regulated, genes modulated later or always were mainly up-regulated. Functional analysis and enrichment of GO terms in DEG sets revealed that processes related to regulation, translation and protein folding, associated with functions such as RNA binding, isomerases and structural molecular activities were affected, indicating that profound changes in the fungal metabolism occurred in OTAI as compared to OTAN conditions.

Up-regulated DEGs included several genes with hydrolase activity in carbohydrate metabolism. Hydrolysis of carbohydrates may contribute to generate malonate and acetate which are substrates used by PKS to generate dihydrocumarin [37]. Moreover, other primary metabolic pathways could be also responsible for generating polyketide precursors. The GO category amino acid metabolism was significantly over-represented among up-regulated genes. The existence of a relationship between mycotoxin biosynthesis (*e*.*g*., aflatoxin and OTA) and amino acid metabolism has been reported in different fungal species. It has been suggested that amino acid catabolism increases intracellular concentration of acetyl-CoA, used as carbon source in polyketide biosynthesis [38]. Some DEGs identified by RNA-Seq were involved in the metabolism of amino acids, such as proline, glutamic and aspartic acids. Proline and glutamic acids in liquid media are known to promote OTA production in *A*. *ochraceus* and *Penicillium viridicatum* [39] and aspartic acid and other amino acids promote aflatoxin production in *Aspergillus* spp. [38]. Furthermore, glutamic acid is incorporated in both the isocoumarin ring and phenylalanine of OTA by *A*. *ochraceus* [40].

Genes up-regulated by OTAI conditions were mainly involved in secondary metabolic processes and the oxidoreductase activity was one of the more represented molecular functions. The two categories included DEGs such as 2 *PAL*, 5 *pks* and 5 *nrps* genes in addition to several methyltransferases, P450 monooxygenases, dehydrogenases, hydrolases and 1 cloroperoxidase, all genes involved in the biosynthesis of mycotoxins and other SMs [14].

RNA-seq analysis identified several DEGs associated with transport and secretion activities, including MFS and ABC transporters, in addition to transporters of other compounds (amino acids, antibiotics, etc.). Transporters, acting as efflux pumps, play a role in avoiding auto-toxic effects by extruding toxic metabolites endogenously produced by micro-organisms [41]. Two MFS transporters (FLU1 and Atr1) putatively involved in OTA transport have been reported in *A*. *carbonarius* [21]. FLU1 (DEG 135554) was always significantly up-regulated in OTAI conditions while Atr1 (DEG 49724) was not differentially expressed in our experimental conditions. The MFS transporter 173225, up-regulated under OTAI conditions, was homologous to the *aflt* gene within the aflatoxin biosynthetic gene cluster in *A*. *flavus* and *A*. *parasiticus* [42]. MFS transporters are involved in the transport of mycotoxins, such as trichothecene and cercosporine, in several fungal species (*e*.*g*., [43]) and the *otatraPN* gene of *P*. *nordicum* is responsible for OTA secretion [44]. The up-regulated ABC transporter 211441 showed homology with the *atrF* gene of *A*. *fumigatus* which is involved in cell drug extrusion [45].

The involvement of PKS and NRPS in mycotoxin biosynthesis has been reported in several fungal species. PKS catalyses reactions leading to the isocoumarin ring and NRPS is responsible for the peptide bond formation between the isocoumarin nucleus and an L-phenylalanine [14]. Comparative RNA-Seq analysis between OTAI and OTAN conditions corroborates previous results. Three *pks* genes (*Acpks1*, *Acpks3* and *Acpks4*) [33] were not differentially expressed, while the *Acpks* (DEG 505925) and *AcOTApks* (173482) genes were significantly up-regulated under OTAI conditions. The latter gene has been proved involved in OTA biosynthetic pathway of *A*. *carbonarius* by gene disruption [17,33]). In addition, 4 novel putative *pks* genes were identified among those up-regulated under OTAI conditions. The *pks* DEG 172075 was homologous to the *Alb1* gene of *A*. *fumigatus* involved in melanin biosynthesis [46]. The two *pks* DEGs, 5570 and 5640, with identical sequences, as well as the *pks* DEG 56260 did not display any sequence homology to previously characterized genes and their functional role remains to be established. RNA-Seq confirmed up-regulation by OTAI conditions of the *AcOTAnrps* gene (DEG 132610), whose role in OTA biosynthesis has been recently demonstrated in *A*. *carbonarius* by gene disruption [19]. A single *cpo* gene (212238), encoding a chloroperoxidase, was significantly up-regulated under OTAI conditions and its expression levels increased during the time. This gene might be responsible for the addition of a chlorine atom to an OTA precursor, in the last step of the OTA biosynthetic pathway, although its role should be demonstrated with specific gene knockout studies. For all the up-regulated *pks*, *nrps* and *cpo* genes, RT-qPCR yielded results consistent with the gene expression patterns revealed by RNA-Seq, thus evidencing the validity of the approach.

A large number of DEGs were involved in SM biosynthesis suggesting that OTAI conditions adopted in our experiments induced not only OTA biosynthesis but also other metabolic pathways. Genes responsible for biosynthesis of SMs in filamentous fungi are frequently in clusters and transcriptionally co-regulated [47]. Clusters often include genes encoding proteins involved in metabolite secretion as well as regulatory proteins containing DNA-binding sequences. For instance, in *A*. *nidulans* a 60-kb genomic region contains a cluster of 25 co-regulated genes needed for the biosynthesis of sterigmatocystin [48]. Organization into gene clusters has been also reported for genes involved in the biosynthesis of aflatoxins in *A*. *flavus* and *A*. *parasiticus* [42], deoxynivalenol and fumonisin in *Fusarium* spp. [49,50]. An OTA gene cluster has been described in *P*. *nordicum* and contains 5 genes: an alkaline serine protease (*asp*PN), a *pks* (*otapks*PN), an *nrps* (*otanps*PN), an MFS transporter (*otatra*PN), responsable for OTA extrusion, and a chloroperoxidase (*otachl*PN) [44].

The availability of assembled sequences of the *A*. *carbonarius* genome and the RNA-Seq data made it possible to investigate gene clusters potentially involved in SM biosynthetic pathway, with particular reference to OTA. Cluster prediction using antiSMASH 2.0 led to identify a large number of putative SM gene clusters in the genome like it occurs in other *Aspergillus* species [51]. QT clustering of RNA-Seq data was then applied to select CEGs in the predicted clusters. This approach, along with gene functional analysis, led to identify 1 gene cluster down-regulated and 14 gene clusters up-regulated under OTAI conditions, encoding enzymatic or regulatory proteins potentially involved in the biosynthesis of mycotoxins or other SMs.

Most of the putative gene clusters identified in this study contain genes encoding enzymes involved in the synthesis of unknown products and are therefore candidates of novel SM pathways. Only the putative clusters 22, 32 and 42 are conserved in the genomes of several species of *Aspergillus* section *Nigri* (S5 Table), while most clusters seem to be unique for the species *A*. *carbonarius*, and hence they might represent sources of exclusive metabolites useful for the specific lifestyle of the fungus.

The cluster 38, including both the *pks* and *nrps* genes involved in the OTA biosynthesis [17,19], contained 6 CEGs in our experimental conditions: i) the *AcOTApks* gene (DEG 173482); ii) the DEG 209537 coding for a hypothetical protein, iii) the *AcOTAnps* gene (DEG 132610), iv) the DEG 517149 encoding a cytochrome P450 monooxygenase (*cyp*), v) the DEG 209543 encoding a monooxygenase containing a FAD-binding domain (*fmo*), vi) the DEG 7821 encoding a bZIP type transcription factor (*bZIP*). Three of the included genes (*pks*, *nrps* and *cyp*) were conserved in other OTA-producer species of *Aspergillus* and *Penicillium* [15,16,17,18,19,34]. With the only exception of the hypothetical protein, homologous genes were found, similarly organized in a gene cluster, in the genome of an OTA-producer strain but not in that of an OTA not-producer strain of *A*. *niger* [52]. All these findings support the hypothesis that the cluster 38 is likely the OTA gene cluster in *A*. *carbonarius*. The cluster includes a bZIP (basic leucine zipper)-type transcription factor (TF). The family of eukaryotic bZIP transcription factors plays critical roles in environmental responses and are associated with production of SMs and in the regulation of mycotoxin biosynthesis in *Aspergillus* species [53].

SM gene clusters often contain a TF regulating specifically gene expression within the cluster [54]; TFs of the *Zn*(2)-*Cys*(6) family, were found in some of the identified gene clusters. A TF of the same type (*aflR*) activates transcription of gene clusters involved in aflatoxin and sterigmatocystin biosynthesis pathway in *A*. *flavus*, *A*. *nidulans* and *A*. *parasiticus* [55,56,57]. SM biosynthesis is responsive to environmental cues, including carbon and nitrogen source, temperature, light, and pH. Different regulation pathways can mediate these environmental signals activating one or more gene clusters [54], and indeed we found 4 putative gene clusters significantly co-regulated with the putative OTA gene cluster. The key genes for these clusters encoded: antibiotic synthetase (cluster 24), NRPS (40), PKS (42) and NRPS-like enzyme (48).

OTAI conditions induced an abundant conidiation and some up-regulated genes were indeed involved in asexual sporulation, while genes involved in sexual sporulation were more represented among down-regulated genes. We identified an up-regulated TF (DEG 212444) having homology with the *brlA* gene, conserved in several *Aspergillus* species, and playing a key role in the activation of conidiogenesis [58,59]. Two *A*. *carbonarius* TFs (*VeA* and *LaeA*) regulating conidiation and OTA biosynthesis in response to light [60] were not modulated under our experimental conditions. Genetic links between SM biosynthesis and fungal development have been widely studied [54]. A connection between mycotoxin biosynthesis, conidiation and ornithine metabolism has been established in aflatoxin-producer *A*. *parasiticus* [61] and proposed for *A*. *carbonarius* [62]. We identified at least 6 highly up-regulated DEGs related to ornitine metabolic pathway: DEGs 399389, 212184 and 507488 encoding enzymes responsible for glutamate production and DEGs 206969, 154116 and 7736 converting glutamate to ornithine. These findings suggest that they can be key genes in early steps of OTA biosynthesis and sporulation in *A*. *carbonarius*, although deeper investigations on the issue are needed.

In conclusion, RNA-Seq results herein discussed represent a comprehensive transcriptional profiling of genes induced under growing conditions promoting OTA biosynthesis using 4 *A*. *carbonarius* strains. Our results are consistent with previous findings, obtained with different approaches, including gene disruption [17,19], suppression subtractive hybridization and proteomic analysis [21,62], and provide further evidence of the involvement of specific OTA-related genes, the activation of major metabolic pathways leading to OTA biosynthesis and their possible connection with other biological processes (*i*.*e*., fungal growth, sporulation and pigmentation).

## Supporting Information

S1 Fig Multi-level pie charts of GO processes for up-regulated (a-c) and down-regulated (d-f) genes of DEG Sets identified by the Venn diagram. (TIF)

S2 Fig Clustering analysis of 890 genes highly up-regulated (FC > 8 and FDR ≤ 0.05) in *A*. *carbonarius* under OTAI *vs*. OTAN conditions, grouped in four clusters (a-d) based on their expression patterns. The numbers in parentheses indicate gene numbers in the cluster. Blue lines are genes listed in Table 1, selected for their potential involvement in OTA production.
(TIF)

S1 Table Primer pairs used in RT-qPCR analysis. (DOC)

S2 Table Statistics of the Illumina 50-bp single reads and mapping on *A*. *carbonarius* transcript and scaffold sequences. (DOC)

S3 Table Correlation matrix between all sample pairwise combinations based on RPKM values. (DOC)

S4 Table Enrichment analysis of GO terms over-represented in DEG sets at each sampling time. (DOC)

S5 Table Prediction of putative gene clusters in *A*. *carbonarius* and co-expression analysis. (XLS)

S6 Table Correlation matrix between the expression of putative gene clusters identified in this study. (DOC)
