# Supplementary figures and images for: RNA-Seq Reveals OTA-Related Gene Transcriptional Changes in Aspergillus carbonarius
Source: PLoS One. 2016 Jan 14;11(1):e0147089. doi: 10.1371/journal.pone.0147089 (PMC4713082; doi:10.1371/journal.pone.0147089)

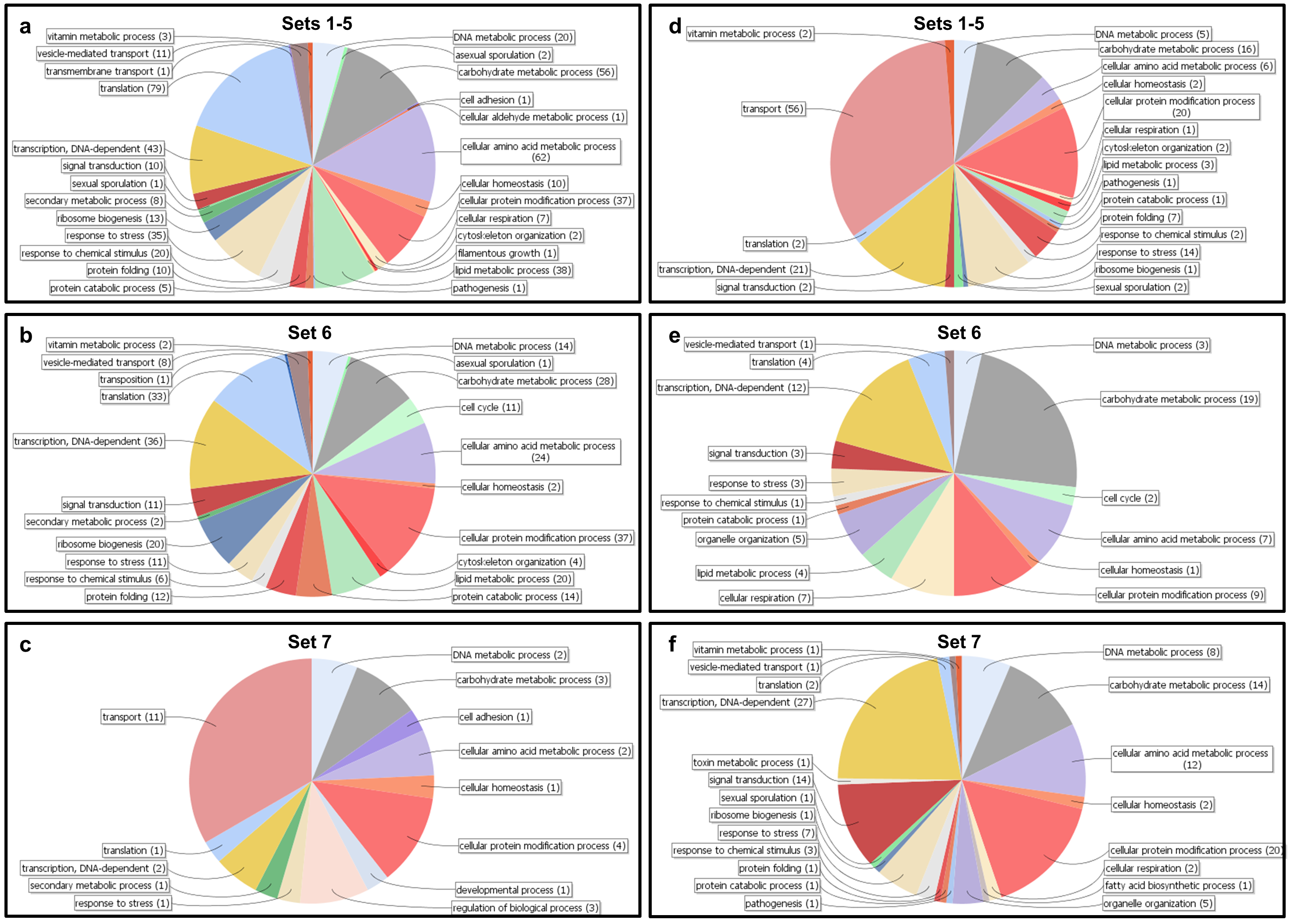

Supplement: S1 Fig — (TIF) [file pone.0147089.s001.tif]

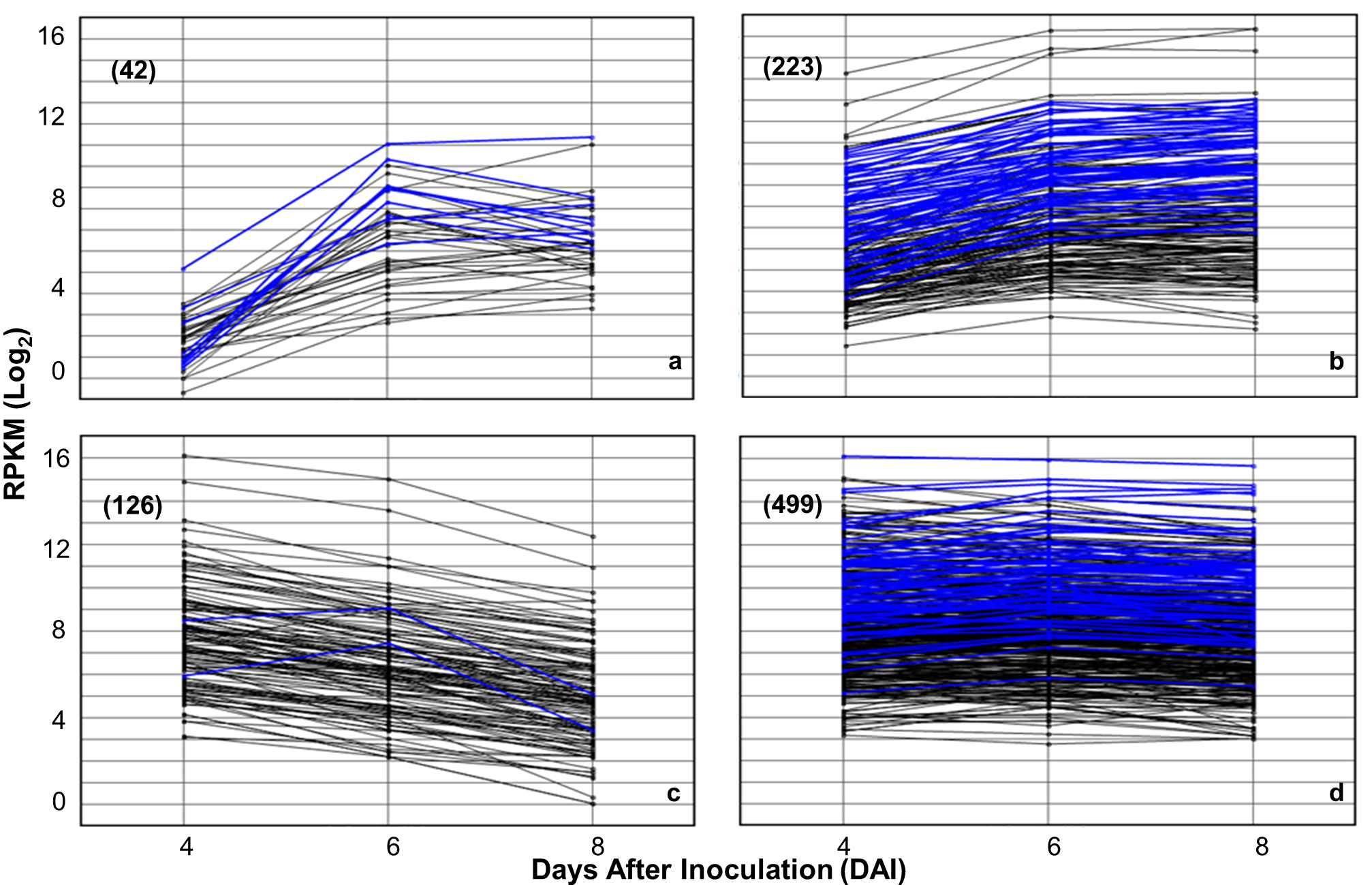

Supplement: S2 Fig — The numbers in parentheses indicate gene numbers in the cluster. Blue lines are genes listed in Table 1, selected for their potential involvement in OTA production. (TIF) [file pone.0147089.s002.tif]
