# Supplementary material for: RNA-Seq Reveals OTA-Related Gene Transcriptional Changes in Aspergillus carbonarius
Source: PLoS One. 2016 Jan 14;11(1):e0147089. doi: 10.1371/journal.pone.0147089 (PMC4713082; doi:10.1371/journal.pone.0147089)
Supplement: S1 Table — (DOC) [file pone.0147089.s003.doc]

**S1 Table.** **Primer pairs used in RT-qPCR analysis.**

| **Target/reference genes** | **Tanscript ID*** | **Primer pairs** | **Sequences (5'-3') Forward / Reverse** | **Amplicon size (bp)** |
| --- | --- | --- | --- | --- |
| Polyketide synthases  (*pks*) | 5570/ 5640 | pks1For/pks1Rev | AAGTGATCTTGCCCGAGATG/ | 211 |
|  |  |  | ACCAGTCCAATCTGCAAAGC |  |
|  | 56260 | pks2For/pks2Rev | TTGGATCAAAGTCTGCCTCC/ | 219 |
|  |  |  | CAGGCAAGGGTCATGAAAGT |  |
|  | 172075 | pks3For/pks3Rev | ACAGCACCAAGAAGGCCACT/ | 211 |
|  |  |  | TGCAAGATACGCTCGATGTC |  |
|  | 173482 | pks4For/pks4Rev | TCTGTATGAGCGCATCGCC/ | 213 |
|  |  |  | GCAGAAGGCCACTTTCCAG |  |
|  | 505925 | pks5For/pks5Rev | TTGTACACCTCGGGAAGCA/ | 156 |
|  |  |  | CACCACCGACAGGTCAAAG |  |
| Non-ribosomal peptide synthases  (*nrps*) | 204544 | nprs2For/nprs2Rev | CATCCTGCAGGCAGAAACAC/ | 164 |
|  |  |  | GCCTGAACCAGATCACCTGT |  |
|  | 209989 | nprs3For/nprs3Rev | AAAACCTACATGGAGCAGGC/ | 225 |
|  |  |  | CACGGGAAGACCAAAGACAT |  |
|  | 505182 | nprs4For/nprs4Rev | TGGTGTCCTGGTTGACCTGA/ | 205 |
|  |  |  | GCGAAACAGATCTTGCCAGT |  |
|  | 132610 | nprs6For/nprs6Rev | GATTCCGATGGAACTGCAAT/ | 196 |
|  |  |  | CTGCCCCAGCATATCAATCT |  |
| Chloroperoxidase (*cpo*) | 212238 | cpx1For/cpx1Rev | AACGATGTGCGAGGGCCTT/ | 171 |
|  |  |  | GTTTGTGGTCAGTGCCTCCT |  |
| β-tubulin  (*β-tub*) | 202852 | tubFor/tubRev | ACTTCAACGAGGCTAGCGG/ | 152 |
|  |  |  | GTTGTTACCAGCACCGGACT |  |
| Calmodulin  (*cal*) | 205510 | calFor/calRev | CTTCCCCGAATTCCTTACC/ | 189 |
|  |  |  | TCACGGATCATCTCATCGAC |  |
| Ubiquitin-coniugating enzyme (*ubc*) | 393986 | ubFw/ubRev | CCGAAGGTCAACTTCACCAC/ | 246 |
|  |  |  | GGCATATTTGCGAGTCCATT |  |
| *Transcript identification number (DOE Joint Genome Institute, http://genome.jgi-psf.org/Aspca3/Aspca3.home.html). | | | | |
