# Supplementary material for: RNA-Seq Reveals OTA-Related Gene Transcriptional Changes in Aspergillus carbonarius
Source: PLoS One. 2016 Jan 14;11(1):e0147089. doi: 10.1371/journal.pone.0147089 (PMC4713082; doi:10.1371/journal.pone.0147089)
Supplement: S2 Table — (DOC) [file pone.0147089.s004.doc]

**S2 Table. Statistics of the Illumina 50-bp single reads and mapping on *A. carbonarius* transcript and scaffold sequences.**

| **Experimental condition*** | **Sampling**  **time**  **(DAI)** | **Strain** | **N. total reads** | **Transcripts** | | | **Scaffolds** | | |
| --- | --- | --- | --- | --- | --- | --- | --- | --- | --- |
|  |  |  |  | **Total mapped**  **reads (%)** | **N. mapped reads** | | **Total mapped**  **reads (%)** | **N. mapped reads** | |
|  |  |  |  |  | **Unique match** | **Multi-position**  **matches** |  | **Unique match** | **Multi-position**  **matches** |
| OTAI | 4 | AC49 | 9,876,538 | 7,911,966 (80.1) | 6,560,055 | 1,351,911 | 9,032,767 (91.5) | 7,840,693 | 1,192,074 |
|  |  | AC66 | 11,509,643 | 10,069,920 (87.5) | 8,388,114 | 1,681,806 | 10,921,534 (94.9) | 10,173,054 | 748,480 |
|  |  | AC67 | 10,345,277 | 8,395,515 (81.2) | 6,978,045 | 1,417,470 | 9,547,109 (92.3) | 8,365,176 | 1,181,933 |
|  |  | AC70 | 10,342,724 | 8,798,249 (85.1) | 7,349,942 | 1,448,307 | 9,718,582 (93.9) | 8,895,328 | 823,254 |
|  | 6 | AC49 | 10,513,633 | 6,899,587 (65.6) | 5,688,939 | 1,210,648 | 9,124,593 (86.8) | 6,508,140 | 2,616,453 |
|  |  | AC66 | 12,750,228 | 10,560,363 (82.8) | 8,661,216 | 1,899,147 | 11,828,801 (92.7) | 10,351,884 | 1,476,917 |
|  |  | AC67 | 10,206,094 | 7,686,585 (75.3) | 6,386,664 | 1,299,921 | 9,172,653 (89.9) | 7,526,021 | 1,646,632 |
|  |  | AC70 | 11,140,068 | 8,779,858 (78.8) | 7,236,713 | 1,543,145 | 10,017,844 (89.9) | 8,680,725 | 1,337,119 |
|  | 8 | AC49 | 9,271,692 | 6,645,881 (71.7) | 5,520,223 | 1,125,658 | 8,261,436 (89.1) | 6,500,283 | 1,761,153 |
|  |  | AC66 | 11,817,931 | 9,240,655 (78.2) | 7,536,438 | 1,704,217 | 10,778,043 (91.2) | 8,984,182 | 1,793,861 |
|  |  | AC67 | 10,650,691 | 8,308,734 (78.0) | 6,945,837 | 1,362,897 | 9,727,661 (91.3) | 8,263,154 | 1,464,507 |
|  |  | AC70 | 10,773,379 | 8,423,096 (78.2) | 6,866,085 | 1,557,011 | 9,849,273 (91.4) | 8,256,234 | 1,593,039 |
| OTAN | 4 | AC49 | 10,923,826 | 9,139,790 (83.7) | 7,574,776 | 1,565,014 | 10,276,547 (94.1) | 9,426,789 | 849,758 |
|  |  | AC66 | 11,120,219 | 9,394,051 (84.5) | 7,687,845 | 1,706,206 | 10,628,288 (95.6) | 9,942,572 | 685,716 |
|  |  | AC67 | 11,481,687 | 9,759,034 (85.0) | 8,086,747 | 1,672,287 | 10,862,019 (94.6) | 10,077,006 | 785,013 |
|  |  | AC70 | 8,898,799 | 7,503,200 (84.3) | 6,185,599 | 1,317,601 | 8,529,422 (95.8) | 7,975,923 | 553,499 |
|  | 6 | AC49 | 10,213,616 | 8,710,621 (85.3) | 7,420,040 | 1,290,581 | 9,620,971 (94.2) | 8,966,685 | 654,286 |
|  |  | AC66 | 12,156,337 | 10,467,486 (86.1) | 8,611,289 | 1,856,197 | 11,603,142 (95.4) | 10,795,396 | 807,746 |
|  |  | AC67 | 9,644,857 | 8,193,001 (84.9) | 6,976,612 | 1,216,389 | 9,038,949 (93.7) | 8,409,512 | 629,437 |
|  |  | AC70 | 10,526,785 | 9,079,805 (86.3) | 7,443,958 | 1,635,847 | 10,077,419 (95.7) | 9,311,920 | 765,499 |
|  | 8 | AC49 | 10,184,028 | 8,423,833 (82.7) | 7,118,291 | 1,305,542 | 9,615,314 (94.4) | 8,874,278 | 741,036 |
|  |  | AC66 | 11,056,015 | 9,685,084 (87.6) | 7,925,948 | 1,759,136 | 10,623,747 (96.1) | 9,890,942 | 732,805 |
|  |  | AC67 | 9,920,503 | 8,043,511 (81.1) | 6,800,126 | 1,243,385 | 9,342,059 (94.2) | 8,444,641 | 897,418 |
|  |  | AC70 | 9,965,775 | 8,471,363 (85.0) | 7,080,100 | 1,391,263 | 9,498,889 (95.3) | 8,767,601 | 731,288 |
| *OTAI: OTA inducing conditions; OTAN: OTA non-inducing conditions. | | | | | | | | | |
