# Supplementary material for: RNA-Seq Reveals OTA-Related Gene Transcriptional Changes in Aspergillus carbonarius
Source: PLoS One. 2016 Jan 14;11(1):e0147089. doi: 10.1371/journal.pone.0147089 (PMC4713082; doi:10.1371/journal.pone.0147089)
Supplement: S3 Table — (DOC) [file pone.0147089.s005.doc]

**S3 Table. Correlation matrix between all sample pairwise combinations based on RPKM values.**

| **Experimental**  **condition*** | | | **Isolate** | **4 DAI** | | | | | | | | | | | | | | | | **6 DAI** | | | | | | | | | | | | | | | | **8 DAI** | | | | | | | | | | | | | | | | |
| --- | --- | --- | --- | --- | --- | --- | --- | --- | --- | --- | --- | --- | --- | --- | --- | --- | --- | --- | --- | --- | --- | --- | --- | --- | --- | --- | --- | --- | --- | --- | --- | --- | --- | --- | --- | --- | --- | --- | --- | --- | --- | --- | --- | --- | --- | --- | --- | --- | --- | --- | --- | --- |
|  |  |  |  | **OTAI** | | | | | | | | **OTAN** | | | | | | | | **OTAI** | | | | | | | | **OTAN** | | | | | | | | **OTAI** | | | | | | | | **OTAN** | | | | | | | | |
|  |  |  |  | 70 | | 66 | | 49 | | 67 | | 70 | | 66 | | 49 | | 67 | | 70 | | 66 | | 49 | | 67 | | 70 | | 66 | | 49 | | 67 | | 70 | | 66 | | 49 | | 67 | | 70 | | 66 | | 49 | | 67 | | |
| **4 DAI** | **OTAN** | 70 | | | ----- | | 0.94 | | 0.89 | | 0.89 | | 0.74 | | 0.71 | | 0.78 | | 0.77 | | 0.87 | | 0.86 | | 0.78 | | 0.80 | | 0.64 | | 0.63 | | 0.66 | | 0.65 | | 0.83 | | 0.81 | | 0.72 | | 0.72 | | 0.57 | | 0.59 | | 0.61 | | 0.60 | |
|  |  | 66 | | | 0.94 | | ----- | | 0.90 | | 0.90 | | 0.74 | | 0.72 | | 0.79 | | 0.78 | | 0.86 | | 0.90 | | 0.79 | | 0.82 | | 0.63 | | 0.63 | | 0.66 | | 0.65 | | 0.83 | | 0.84 | | 0.74 | | 0.75 | | 0.55 | | 0.58 | | 0.61 | | 0.60 | |
|  |  | 49 | | | 0.89 | | 0.90 | | ----- | | 0.97 | | 0.66 | | 0.63 | | 0.76 | | 0.77 | | 0.88 | | 0.88 | | 0.83 | | 0.88 | | 0.57 | | 0.56 | | 0.63 | | 0.63 | | 0.83 | | 0.82 | | 0.77 | | 0.79 | | 0.51 | | 0.53 | | 0.62 | | 0.60 | |
|  |  | 67 | | | 0.89 | | 0.90 | | 0.97 | | ----- | | 0.65 | | 0.62 | | 0.76 | | 0.76 | | 0.88 | | 0.89 | | 0.85 | | 0.89 | | 0.57 | | 0.56 | | 0.63 | | 0.63 | | 0.83 | | 0.83 | | 0.79 | | 0.81 | | 0.52 | | 0.53 | | 0.63 | | 0.61 | |
|  | **OTAI** | 70 | | | 0.74 | | 0.74 | | 0.66 | | 0.65 | | ----- | | 0.91 | | 0.80 | | 0.75 | | 0.65 | | 0.67 | | 0.63 | | 0.65 | | 0.82 | | 0.82 | | 0.75 | | 0.75 | | 0.69 | | 0.66 | | 0.59 | | 0.60 | | 0.70 | | 0.75 | | 0.64 | | 0.64 | |
|  |  | 66 | | | 0.71 | | 0.72 | | 0.63 | | 0.62 | | 0.91 | | ----- | | 0.79 | | 0.72 | | 0.63 | | 0.65 | | 0.60 | | 0.62 | | 0.81 | | 0.84 | | 0.73 | | 0.73 | | 0.66 | | 0.64 | | 0.58 | | 0.58 | | 0.70 | | 0.76 | | 0.63 | | 0.64 | |
|  |  | 49 | | | 0.78 | | 0.79 | | 0.76 | | 0.76 | | 0.80 | | 0.79 | | ----- | | 0.95 | | 0.73 | | 0.74 | | 0.70 | | 0.73 | | 0.75 | | 0.75 | | 0.78 | | 0.78 | | 0.73 | | 0.71 | | 0.69 | | 0.71 | | 0.63 | | 0.70 | | 0.74 | | 0.74 | |
|  |  | 67 | | | 0.77 | | 0.78 | | 0.77 | | 0.76 | | 0.75 | | 0.72 | | 0.95 | | ----- | | 0.72 | | 0.73 | | 0.68 | | 0.71 | | 0.66 | | 0.68 | | 0.72 | | 0.72 | | 0.72 | | 0.69 | | 0.68 | | 0.69 | | 0.54 | | 0.62 | | 0.70 | | 0.69 | |
| **6 DAI** | **OTAN** | 70 | | | 0.87 | | 0.86 | | 0.88 | | 0.88 | | 0.65 | | 0.63 | | 0.73 | | 0.72 | | ----- | | 0.91 | | 0.85 | | 0.89 | | 0.59 | | 0.58 | | 0.63 | | 0.63 | | 0.94 | | 0.85 | | 0.80 | | 0.81 | | 0.54 | | 0.56 | | 0.63 | | 0.61 | |
|  |  | 66 | | | 0.86 | | 0.90 | | 0.88 | | 0.89 | | 0.67 | | 0.65 | | 0.74 | | 0.73 | | 0.91 | | ----- | | 0.87 | | 0.90 | | 0.61 | | 0.61 | | 0.67 | | 0.66 | | 0.89 | | 0.94 | | 0.83 | | 0.86 | | 0.57 | | 0.59 | | 0.66 | | 0.64 | |
|  |  | 49 | | | 0.78 | | 0.79 | | 0.83 | | 0.85 | | 0.63 | | 0.60 | | 0.70 | | 0.68 | | 0.85 | | 0.87 | | ----- | | 0.96 | | 0.61 | | 0.59 | | 0.69 | | 0.68 | | 0.88 | | 0.89 | | 0.94 | | 0.93 | | 0.62 | | 0.58 | | 0.71 | | 0.69 | |
|  |  | 67 | | | 0.80 | | 0.82 | | 0.88 | | 0.89 | | 0.65 | | 0.62 | | 0.73 | | 0.71 | | 0.89 | | 0.90 | | 0.96 | | ----- | | 0.62 | | 0.60 | | 0.70 | | 0.69 | | 0.90 | | 0.90 | | 0.91 | | 0.92 | | 0.61 | | 0.59 | | 0.71 | | 0.69 | |
|  | **OTAI** | 70 | | | 0.64 | | 0.63 | | 0.57 | | 0.57 | | 0.82 | | 0.81 | | 0.75 | | 0.66 | | 0.59 | | 0.61 | | 0.61 | | 0.62 | | ----- | | 0.93 | | 0.86 | | 0.86 | | 0.63 | | 0.63 | | 0.60 | | 0.61 | | 0.88 | | 0.90 | | 0.74 | | 0.75 | |
|  |  | 66 | | | 0.63 | | 0.63 | | 0.56 | | 0.56 | | 0.82 | | 0.84 | | 0.75 | | 0.68 | | 0.58 | | 0.61 | | 0.59 | | 0.60 | | 0.93 | | ----- | | 0.84 | | 0.85 | | 0.61 | | 0.63 | | 0.58 | | 0.58 | | 0.83 | | 0.92 | | 0.71 | | 0.72 | |
|  |  | 49 | | | 0.66 | | 0.66 | | 0.63 | | 0.63 | | 0.75 | | 0.73 | | 0.78 | | 0.72 | | 0.63 | | 0.67 | | 0.69 | | 0.70 | | 0.86 | | 0.84 | | ----- | | 0.97 | | 0.68 | | 0.69 | | 0.70 | | 0.72 | | 0.83 | | 0.84 | | 0.88 | | 0.88 | |
|  |  | 67 | | | 0.65 | | 0.65 | | 0.63 | | 0.63 | | 0.75 | | 0.73 | | 0.78 | | 0.72 | | 0.63 | | 0.66 | | 0.68 | | 0.69 | | 0.86 | | 0.85 | | 0.97 | | ----- | | 0.67 | | 0.68 | | 0.69 | | 0.70 | | 0.83 | | 0.84 | | 0.85 | | 0.86 | |
| **8 DAI** | **OTAN** | 70 | | | 0.83 | | 0.83 | | 0.83 | | 0.83 | | 0.69 | | 0.66 | | 0.73 | | 0.72 | | 0.94 | | 0.89 | | 0.88 | | 0.90 | | 0.63 | | 0.61 | | 0.68 | | 0.67 | | ----- | | 0.87 | | 0.84 | | 0.84 | | 0.60 | | 0.61 | | 0.68 | | 0.66 | |
|  |  | 66 | | | 0.81 | | 0.84 | | 0.82 | | 0.83 | | 0.66 | | 0.64 | | 0.71 | | 0.69 | | 0.85 | | 0.94 | | 0.89 | | 0.90 | | 0.63 | | 0.63 | | 0.69 | | 0.68 | | 0.87 | | ----- | | 0.86 | | 0.88 | | 0.62 | | 0.62 | | 0.68 | | 0.66 | |
|  |  | 49 | | | 0.72 | | 0.74 | | 0.77 | | 0.79 | | 0.59 | | 0.58 | | 0.69 | | 0.68 | | 0.80 | | 0.83 | | 0.94 | | 0.91 | | 0.60 | | 0.58 | | 0.70 | | 0.69 | | 0.84 | | 0.86 | | ----- | | 0.96 | | 0.61 | | 0.57 | | 0.76 | | 0.74 | |
|  |  | 67 | | | 0.72 | | 0.75 | | 0.79 | | 0.81 | | 0.60 | | 0.58 | | 0.71 | | 0.69 | | 0.81 | | 0.86 | | 0.93 | | 0.92 | | 0.61 | | 0.58 | | 0.72 | | 0.70 | | 0.84 | | 0.88 | | 0.96 | | ----- | | 0.61 | | 0.58 | | 0.76 | | 0.74 | |
|  | **OTAI** | 70 | | | 0.57 | | 0.55 | | 0.51 | | 0.52 | | 0.70 | | 0.70 | | 0.63 | | 0.54 | | 0.54 | | 0.57 | | 0.62 | | 0.61 | | 0.88 | | 0.83 | | 0.83 | | 0.83 | | 0.60 | | 0.62 | | 0.61 | | 0.61 | | ----- | | 0.87 | | 0.74 | | 0.75 | |
|  |  | 66 | | | 0.59 | | 0.58 | | 0.53 | | 0.53 | | 0.75 | | 0.76 | | 0.70 | | 0.62 | | 0.56 | | 0.59 | | 0.58 | | 0.59 | | 0.90 | | 0.92 | | 0.84 | | 0.84 | | 0.61 | | 0.62 | | 0.57 | | 0.58 | | 0.87 | | ----- | | 0.71 | | 0.72 | |
|  |  | 49 | | | 0.61 | | 0.61 | | 0.62 | | 0.63 | | 0.64 | | 0.63 | | 0.74 | | 0.70 | | 0.63 | | 0.66 | | 0.71 | | 0.71 | | 0.74 | | 0.71 | | 0.88 | | 0.85 | | 0.68 | | 0.68 | | 0.76 | | 0.76 | | 0.74 | | 0.71 | | ----- | | 0.97 | |
|  |  | 67 | | | 0.60 | | 0.60 | | 0.60 | | 0.61 | | 0.64 | | 0.64 | | 0.74 | | 0.69 | | 0.61 | | 0.64 | | 0.69 | | 0.69 | | 0.75 | | 0.72 | | 0.88 | | 0.86 | | 0.66 | | 0.66 | | 0.74 | | 0.74 | | 0.75 | | 0.72 | | 0.97 | | ----- | |
| *DAI: Days After Inoculation; OTAI: OTA inducing conditions; OTAN: OTA non-inducing conditions. | | | | | | | | | | | | | | | | | | | | | | | | | | | | | | | | | | | | | | | | | | | | | | | | | | | |  |
