# Supplementary material for: RNA-Seq Reveals OTA-Related Gene Transcriptional Changes in Aspergillus carbonarius
Source: PLoS One. 2016 Jan 14;11(1):e0147089. doi: 10.1371/journal.pone.0147089 (PMC4713082; doi:10.1371/journal.pone.0147089)
Supplement: S4 Table — (DOC) [file pone.0147089.s006.doc]

**S4 Table. Enrichment analysis of GO terms over-represented in DEG sets at each sampling time.**

| **GO Term** | **Name** | **Type*** | **FDR** | ***p*-value** | **No. of genes** | | | |
| --- | --- | --- | --- | --- | --- | --- | --- | --- |
|  |  |  |  |  | **annotated** | | **non annotated** | |
|  |  |  |  |  | **DEG set** | **Ref**** | **DEG set** | **Ref** |
| **Gene set: 4 DAI** | | | | | | | | |
| *up-regulated* | | | | | | | | |
| GO:0005576 | extracellular region | C | 5.00E-03 | 1.00E-03 | 15 | 119 | 364 | 7,904 |
| GO:0005618 | cell wall | C | 1.20E-02 | 2.50E-03 | 5 | 17 | 374 | 8,006 |
| GO:0005829 | cytosol | C | 1.30E-01 | 3.30E-02 | 5 | 35 | 374 | 7,988 |
| GO:0004030 | aldehyde dehydrogenase [NAD(P)+] activity | F | 1.70E-01 | 4.50E-02 | 1 | 0 | 378 | 8,023 |
| GO:0019748 | secondary metabolic process | P | 1.90E-03 | 3.00E-04 | 7 | 23 | 372 | 8,000 |
| GO:0005975 | carbohydrate metabolic process | P | 9.80E-03 | 2.00E-03 | 32 | 376 | 347 | 7,647 |
| GO:0006081 | cellular aldehyde metabolic process | P | 1.70E-01 | 4.50E-02 | 1 | 0 | 378 | 8,023 |
| *down-regulated* | | | | | | | | |
| GO:0016020 | membrane | C | 7.30E-03 | 1.80E-04 | 70 | 1,539 | 172 | 6,621 |
| GO:0005215 | transporter activity | F | 6.40E-03 | 2.00E-05 | 36 | 563 | 206 | 7,597 |
| GO:0006810 | transport | P | 7.30E-03 | 2.60E-04 | 58 | 1,218 | 184 | 6,942 |
| GO:0034293 | sexual sporulation | P | 2.00E-01 | 3.80E-02 | 2 | 9 | 240 | 8,151 |
| **Gene set: 6 DAI** | | | | | | | | |
| *up-regulated* | | | | | | | | |
| GO:0005840 | ribosome | C | 6.10E-07 | 5.00E-09 | 57 | 114 | 1,241 | 6,990 |
| GO:0005198 | structural molecule activity | F | 1.50E-06 | 1.90E-08 | 54 | 110 | 1,244 | 6,994 |
| GO:0016491 | oxidoreductase activity | F | 1.40E-04 | 7.70E-06 | 288 | 1,201 | 1,010 | 5,903 |
| GO:0016853 | isomerase activity | F | 1.30E-02 | 1.60E-03 | 39 | 117 | 1,259 | 6,987 |
| GO:0003723 | RNA binding | F | 3.70E-02 | 6.60E-03 | 59 | 216 | 1,239 | 6,888 |
| GO:0006412 | translation | P | 3.70E-06 | 5.90E-08 | 85 | 227 | 1,213 | 6,877 |
| GO:0006457 | protein folding | P | 3.10E-03 | 2.80E-04 | 21 | 41 | 1,277 | 7,063 |
| GO:0006520 | cellular amino acid metabolic process | P | 1.60E-02 | 2.30E-03 | 71 | 257 | 1,227 | 6,847 |
| GO:0042254 | ribosome biogenesis | P | 1.60E-01 | 3.70E-02 | 19 | 58 | 1,279 | 7,046 |
| GO:0019748 | secondary metabolic process | P | 1.60E-01 | 3.90E-02 | 9 | 21 | 1,289 | 7,083 |
| *down-regulated* | | | | | | | | |
| GO:0016020 | membrane | C | 8.90E-02 | 7.30E-03 | 171 | 1,438 | 575 | 6,218 |
| GO:0005777 | peroxisome | C | 2.80E-01 | 3.30E-02 | 6 | 22 | 740 | 7,634 |
| GO:0005576 | extracellular region | C | 3.50E-01 | 4.40E-02 | 19 | 115 | 727 | 7,541 |
| GO:0005215 | transporter activity | F | 6.30E-02 | 4.50E-03 | 73 | 526 | 673 | 7,130 |
| GO:0004871 | signal transducer activity | F | 2.20E-01 | 2.40E-02 | 10 | 43 | 736 | 7,613 |
| GO:0016788 | hydrolase activity, acting on ester bonds | F | 2.80E-01 | 3.10E-02 | 24 | 152 | 722 | 7,504 |
| GO:0045333 | cellular respiration | P | 1.00E-01 | 1.00E-02 | 9 | 33 | 737 | 7,623 |
| **Gene set: 8 DAI** | | | | | | | | |
| *up-regulated* | | | | | | | | |
| GO:0016491 | oxidoreductase activity | F | 1.60E-06 | 5.00E-08 | 69 | 1420 | 138 | 6,775 |
| *down-regulated* | | | | | | | | |
| GO:0016020 | membrane | C | 2.00E-01 | 1.40E-03 | 65 | 1,544 | 171 | 6,622 |
| GO:0005576 | extracellular region | C | 2.00E-01 | 4.40E-03 | 10 | 124 | 226 | 8,042 |
| GO:0005215 | transporter activity | F | 2.00E-01 | 4.30E-03 | 29 | 570 | 207 | 7,596 |
| GO:0004672 | protein kinase activity | F | 4.40E-01 | 4.70E-02 | 12 | 229 | 224 | 7,937 |
| GO:0005975 | carbohydrate metabolic process | P | 2.00E-01 | 3.10E-03 | 22 | 386 | 214 | 7,780 |
| GO:0006810 | transport | P | 2.00E-01 | 3.10E-03 | 53 | 1,223 | 183 | 6,943 |
| GO:0045333 | cellular respiration | P | 4.00E-01 | 2.90E-02 | 4 | 38 | 232 | 8,128 |
| GO:0034293 | sexual sporulation | P | 4.00E-01 | 3.70E-02 | 2 | 9 | 234 | 8,157 |
| GO:0009405 | pathogenesis | P | 4.60E-01 | 5.00E-02 | 2 | 11 | 234 | 8,155 |
| *C: cellular component; F: molecular function; P: biological process. **reference transcriptome. | | | | | | | | |
