# Supplementary material for: RNA-Seq Reveals OTA-Related Gene Transcriptional Changes in Aspergillus carbonarius
Source: PLoS One. 2016 Jan 14;11(1):e0147089. doi: 10.1371/journal.pone.0147089 (PMC4713082; doi:10.1371/journal.pone.0147089)
Supplement: S6 Table — (DOC) [file pone.0147089.s008.doc]

**S6 Table. Correlation matrix between the expression of putative gene clusters identified in this study.**

|  |  | Putative gene clusters | | | | | | | | | | | | | |
| --- | --- | --- | --- | --- | --- | --- | --- | --- | --- | --- | --- | --- | --- | --- | --- |
|  |  | 4 | 13 | 14 | 17 | 19 | 22 | 24 | 25 | 32 | 37 | 38 | 40 | 42 | 48 |
| Putative gene clusters | 4 |  | **0.97** | **0.97** | -0.34 | **0.94** | **0.88** | **0.76** | **0.86** | **0.80** | **0.96** | 0.19 | **0.28** | 0.50 | 0.44 |
|  | 13 | **0.97** |  | **0.93** | -0.25 | **0.86** | **0.77** | 0.63 | **0.76** | 0.68 | **0.88** | 0.03 | 0.09 | 0.38 | 0.29 |
|  | 14 | **0.97** | **0.93** |  | -0.37 | **0.97** | **0.94** | **0.86** | **0.94** | **0.90** | **0.97** | 0.37 | 0.40 | 0.66 | 0.54 |
|  | 17 | -0.34 | -0.25 | -0.37 |  | -0.37 | -0.60 | -0.63 | -0.47 | -0.53 | -0.43 | -0.60 | -0.77 | -0.43 | -0.78 |
|  | 19 | **0.94** | **0.86** | **0.97** | -0.37 |  | **0.94** | **0.88** | **0.98** | **0.94** | **0.99** | 0.45 | 0.46 | **0.76** | 0.54 |
|  | 22 | **0.88** | **0.77** | **0.94** | -0.60 | **0.94** |  | **0.97** | **0.97** | **0.97** | **0.95** | 0.62 | 0.69 | **0.76** | **0.78** |
|  | 24 | **0.76** | 0.63 | **0.86** | -0.63 | **0.88** | **0.97** |  | **0.95** | **0.98** | **0.87** | **0.78** | **0.81** | **0.85** | **0.86** |
|  | 25 | **0.86** | **0.76** | **0.94** | -0.47 | **0.98** | **0.97** | **0.95** |  | **0.99** | **0.96** | 0.63 | 0.62 | **0.87** | 0.66 |
|  | 32 | **0.80** | 0.68 | **0.90** | -0.53 | **0.94** | **0.97** | **0.98** | **0.99** |  | **0.92** | 0.73 | 0.71 | **0.90** | 0.74 |
|  | 37 | **0.96** | **0.88** | **0.97** | -0.43 | **0.99** | **0.95** | **0.87** | **0.96** | **0.92** |  | 0.41 | 0.45 | 0.72 | 0.54 |
|  | 38 | 0.19 | 0.03 | 0.37 | -0.60 | 0.45 | 0.62 | **0.78** | 0.63 | 0.73 | 0.41 |  | **0.94** | **0.85** | **0.83** |
|  | 40 | 0.28 | 0.09 | 0.40 | -0.77 | 0.46 | 0.69 | **0.81** | 0.62 | 0.71 | 0.45 | **0.94** |  | 0.71 | **0.95** |
|  | 42 | 0.50 | 0.38 | 0.66 | -0.43 | **0.76** | **0.76** | **0.85** | **0.87** | **0.90** | 0.72 | **0.85** | 0.71 |  | 0.62 |
|  | 48 | 0.44 | 0.29 | 0.54 | -0.78 | 0.54 | **0.78** | **0.86** | 0.66 | 0.74 | 0.54 | **0.83** | **0.95** | 0.62 |  |

Analysis settings: average of RPKM values for each putative gene cluster was used. Bold: correlation higher than 0.75. Light blue: putative gene clusters significantly co-regulated with the putative OTA gene cluster.
